# Young adults in times of overlapping social crises: dynamic profiles of mental health and crisis-related concerns using latent transition analysis

**DOI:** 10.1186/s12992-026-01199-8

**Published:** 2026-02-27

**Authors:** Małgorzata Gambin, Tomasz Oleksy, Anna Wnuk, Izabela Lassota

**Affiliations:** Faculty of Psychology, University of Warsaw, ul. Stawki 5/7, Warsaw, 00-183 Poland

**Keywords:** Mental health, Depression, Anxiety, Resilience, Polycrisis, Emerging adulthood, Latent transition analysis

## Abstract

In recent years, young adults have navigated multiple, simultaneous crises - COVID-19, war in Ukraine, economic turbulence, climate change, and rapid AI growth - which pose complex mental-health risks. Drawing on multisystemic resilience models and the dual-factor model of mental health, we examine how individual (emotion-regulation difficulties), relational (attachment, social support), and contextual resources (social engagement, place attachment, socioeconomic status) relate to distinct emotional-response profiles and their change across three waves (July 2023, February 2024, September 2024) in a representative Polish sample (*N* = 1,110; 51.1% women, 48.5% men, 0.5% another/undisclosed; ages 18–30; M = 24.32, SD = 3.74). Anxiety, depression, life satisfaction, and crisis-related concerns were analysed with LPA and LTA, alongside predictors: emotion-regulation difficulties, attachment anxiety/avoidance, social support, social engagement, place attachment, and socioeconomic indicators. A five-profile solution fit best at each wave, and Wave 1 profile proportions were as follows: (1) *Severely Distressed & Highly Concerned* (14.5%), (2) *Highly Distressed & Moderately Concerned* (26.8%), (3) *Highly Worried but Satisfied* (24.9%), (4) *Content & Mildly Concerned* (30.2%), and (5) *Content & Carefree* (3.6%) with most participants remaining in the same profile across waves. Emotion regulation difficulties and attachment anxiety increased the likelihood of membership in highly distressed profiles. In contrast, lower attachment avoidance, higher social support, and place identity facilitated adaptive transitions toward profiles characterized by lower distress or recovery. Active place attachment and social engagement were distinctly associated with membership in the subgroup marked by high crisis-related concerns but preserved well-being. These person-centered findings reveal diverse, dynamic mental-health trajectories in times of polycrisis. Interventions that foster emotion regulation skills, secure attachment, supportive social networks, and community engagement may help young people maintain or regain well-being amid ongoing societal challenges.

**Supplementary Information:**

The online version contains supplementary material available at 10.1186/s12992-026-01199-8.

## Introduction

Since the early 2000s, research has shown a notable increase in depressive and anxiety symptoms among adolescents and emerging adults in Poland and worldwide, particularly among young women and girls [1–3]. The COVID-19 pandemic has further exacerbated global mental health challenges by disrupting routines, limiting peer interactions, and creating prolonged uncertainty about health and education [4–7]. Pandemic impacts in Central and Eastern Europe - already strained by inflation and labour- and housing-market instability - have been further compounded by the war in Ukraine, refugee influx, democratic challenges, climate change and rapid AI growth [8–12]. This confluence of threats is increasingly characterized as a global polycrisis, a state defined by the causal entanglement of risks across multiple macroregional systems [12]. Unlike discrete co-occurring stressors, which imply temporary coincidence, a polycrisis is theorized to produce emergent properties where crises in one system (e.g., geopolitical conflict) trigger domino effects or inter-systemic feedbacks in others (e.g., economic turmoil or ecological degradation), creating a qualitatively new landscape of risk. For young adults, this entanglement results in multiplied vulnerability, where the psychological impact is not merely a sum of individual stressors but a complex state of cumulative stress [11, 12].

These pressures are especially concerning in emerging adulthood (ages 18–30), a developmental period marked by identity exploration, relationship formation and major educational and vocational choices [13, 14]. Such future-oriented tasks make young adults particularly sensitive to prolonged uncertainty and social disruption. Moreover, reliance on peer relationships and expanding social networks for emotional support makes interpersonal and contextual resources particularly consequential for well-being during this phase [15]. Recent findings among Polish youth show that crisis-related concerns are strongly intertwined with chronic mental-health symptoms [9], further highlighting the need to understand diverse patterns of functioning in this population. While person-centered designs are increasingly utilized, many studies still rely on variable-centered methods that may overlook the individual heterogeneity of emotional responses. Building on these precedents, we address the specific gap in research simultaneously integrating distress and well-being with crises-related concerns. To address this gap, the present study employs a person-centered approach - using Latent Profile Analysis (LPA) and Latent Transition Analysis (LTA) across three waves of data collection - to identify distinct profiles of emotional responses among young adults. By integrating measures of anxiety, depression, life satisfaction, and crisis-related concerns, we seek to uncover nuanced subgroups that may be overlooked by traditional methods. Furthermore, the present study examines predictors of profile membership and transitions - such as emotion regulation, interpersonal attachment, place attachment, social support and engagement, and socioeconomic status - to better understand the factors driving resilience and vulnerability.

### Heterogeneity in mental health responses and limitations of traditional approaches

Several studies have examined mental health across different age groups during and after the pandemic in the context of overlapping social crises. However, most of these studies have been cross-sectional [16, 17], and even longitudinal research has primarily focused on mean-level data. Such approaches may overlook the inherent heterogeneity in individuals’ responses, leading to potentially erroneous conclusions that symptoms and distress are uniform across various stages of the pandemic and other crises [4, 18]. It is now well established that human responses to major stressors are highly variable [19, 20]. A small number of prototypical trajectories typically capture this variability, with resilience (i.e., maintaining stable mental health despite adversity) emerging as the most common pattern [19–21]. During the pandemic, trajectory-based studies have consistently identified resilience in about two-thirds of individuals, while smaller groups experienced chronic distress, delayed symptoms, or recovery. Notably, evidence suggests that a recovery trajectory was less common during COVID-19, with a mild-to-moderate distress pattern emerging more frequently than in pre-pandemic studies - likely reflecting the prolonged and pervasive nature of pandemic-related stressors [20]. Thus, it seems that although resilience is often the most prevalent trajectory following acute stressors, prolonged or recurrent crises may gradually erode coping resources, reducing the likelihood of maintaining psychological stability over time.

A recent longitudinal study from Germany [22] further indicates that young people’s mental health is increasingly shaped by overlapping global crises, including the pandemic, war, economic instability, climate change, and rapid technological transformation. The authors found that while youth mental health partially rebounded after the acute pandemic period, about one in five children and adolescents continued to report low quality of life, anxiety, and mental health problems in late 2023. Importantly, crisis-related future anxiety emerged as a robust predictor of well-being and mental health. At the same time, rapid technological change may constitute an additional source of psychological strain: AI adoption and related technostress have been linked to elevated job stress and burnout, and to poorer mental health outcomes [23–25]. AI has also been discussed in terms of a broader “crisis of work,” capturing concerns about how automation may reshape education-to-work pathways and early-career prospects [26]. Although AI-related anxiety may currently appear less salient than immediate threats such as war or inflation, it may still represent a meaningful stressor for young adults navigating key educational and vocational milestones. In particular, the accelerating automation of work and the potential displacement of entry-level roles [27] may contribute to longer-term insecurity that compounds ongoing economic pressures. This is echoed by the salient distress associated with inflation, which has been shown to be positively related to depressive and anxiety symptoms [28, 29]. Meanwhile, climate anxiety continues to weigh heavily on youth, with nearly half of young people worldwide reporting that climate-related concerns negatively impact daily functioning [30]. Building on these findings, the present study adopts a polycrisis perspective to examine resilience in the face of chronic and compounding stressors beyond the single-crisis focus of prior research.

### Person-centered approaches in longitudinal resilience research

Many longitudinal studies on resilience in the face of social crises have either examined anxiety and depression independently - overlooking their frequent comorbidity - or focused solely on psychopathology without considering dimensions of subjective well-being, such as life satisfaction. The dual-factor model (DFM) of mental health addresses this limitation by accounting for both negative indicators (e.g., anxiety, depression) and positive indicators (e.g., life satisfaction), conceptualizing them as distinct but interrelated dimensions ([21, 31–33]). A central debate within this framework concerns the independence versus interdependence of these dimensions. While traditional models view distress and well-being as opposite ends of a single continuum, the DFM posits that they occupy separate spaces, allowing for identification of complex profiles, such as individuals experiencing high distress along with high well-being. However, these dimensions are often interdependent in practice: high subjective well-being can act as a buffer, preventing high levels of concern or mild symptoms from developing into chronic psychopathology. The present study expands this framework by incorporating a third dimension: crisis-related concerns. We argue that in the context of a global polycrisis, internal emotional states cannot be fully understood in isolation from individuals’ appraisals of systemic threats. By adding this third axis, we move beyond a decontextualized view of mental health to capture the functional interdependence between personal well-being, clinical symptoms, and the psychological internalization of macro-level crises. This tri-dimensional approach is theoretically essential for identifying “Concerned Resilient” profiles - individuals who maintain high life satisfaction despite profound awareness of and concerns about societal crises.

To examine psychological functioning over time, Latent Transition Analysis (LTA) serves as a person-centered model for longitudinal data. As an extension of Latent Class or Latent Profile Analysis, LTA enables researchers to identify unobserved subgroups or “profiles” based on patterns of responses and to estimate how individuals transition between these profiles across multiple time points [32, 34–36].

This person-centered approach is particularly valuable in the context of overlapping and compounding social crises, where individuals’ responses may vary significantly depending on their risk and protective factors. For example, Veijonaho et al. [37] found that Finnish adolescents’ climate-distress and denialism profiles fluctuated markedly over a year, whereas Rzeszutek et al. [38] observed stable well-being profiles in Polish adults facing the dual pressures of post-pandemic stress and war-related economic crisis. Notably, poorer financial conditions predicted membership in the lowest well-being profiles, even after accounting for personality traits.

### The multisystemic nature of resilience: interplay between individual and contextual factors

Research on resilience highlights that it is not merely an individual trait but rather emerges from the interplay of personal and contextual factors in shaping mental health outcomes after stressful events and social crises. Although the specific contributors to resilience vary according to context and stressor characteristics [20], a robust body of research has identified several universal protective factors spanning multiple systems, including individual, family, school, community, and organizational levels [39]. These factors encompass among others: close relationships, social support, self-regulation, and positive perceptions of self, family, or group, as well as a sense of belonging and cohesion that may stem from emotional bonds with communities and significant places. Resilience emerges from dynamic multisystem interactions [20, 39]. This aligns with Bronfenbrenner’s ecological systems theory [40] which situates development within interrelated layers, from the immediate microsystem (family, peers, school) to the broader sociocultural and economic forces of the macrosystem. In this ecological perspective, resilience can be understood as emerging from dynamic processes across levels: macro-level stressors shape the demands placed on families, peers, and communities, while strengths in micro- and mesosystem contexts (e.g., supportive relationships, social support, and place-based bonds) may buffer the psychological impact of these stressors and help sustain individual coping resources over time.

At the individual level, emotion regulation plays a central role in psychological well-being and is widely recognized as a transdiagnostic factor underlying various forms of psychopathology, particularly anxiety and depressive disorders, especially during times of social crises [8, 41–43]. It encompasses the capacity not only to acknowledge and accept one’s own and others’ negative emotions but also to effectively modulate emotional responses in the face of stress. By employing adaptive strategies, individuals can regulate behaviors when distressed, maintain goal-directed actions even during periods of emotional upheaval, and ultimately reduce the intensity and duration of negative affect [44].

However, its efficacy is functionally dependent on the relational and contextual systems in which the individual is embedded. Attachment anxiety and avoidance are central interpersonal attachment dimensions that shape both relationship dynamics and stress responses, including reactions to societal threats [45–48]. A growing body of evidence shows that attachment security fosters adaptive emotion regulation, coping, and recovery under stress, whereas insecure attachment undermines these pathways [45, 49, 50]. Individuals high in attachment anxiety typically rely on hyperactivating strategies: they are hypervigilant to rejection, tend to magnify threats, and seek reassurance in maladaptive ways, often showing intense emotional reactivity. Those high in attachment avoidance use deactivating strategies, leaning toward self-reliance and emotional suppression, which may initially buffer distress but can lead to withdrawal from potential support, foster isolation, and limit opportunities for vulnerability and intimacy. Such patterns can hinder collective coping and social trust during large-scale crises, where interpersonal coordination and perceived security are crucial for maintaining well-being [39]. Accordingly, attachment anxiety and avoidance capture key individual differences in how young adults appraise, manage, and recover from chronic societal stressors, linking relational regulation processes to multisystemic resilience.

Social support further contributes to resilience by buffering against stress through the receipt of emotional, informational, and tangible assistance from family, friends, or communities [51, 52]. Complementing these factors, social engagement, defined as active participation in community and political life, fosters connectedness, a sense of belonging, and collective efficacy. Social engagement—active participation in community and political life—may foster connectedness, belonging, and collective efficacy. Drawing on the Social Identity Model of Collective Action (SIMCA; [53]) and transactional coping theory [54], we propose that crisis-related concerns (i.e. threat appraisals) are most likely to translate into adaptive collective action when accompanied by a salient social identity and perceived collective efficacy. In this context, engagement can transform macro-level threats into goal-directed agency and reciprocal support, functioning as a form of meaning- and problem-focused coping that may help sustain well-being during prolonged crises [55]. 

Place attachment, an emotional bond with places [56, 57] is linked to greater well-being [58, 59], fosters a sense of belonging and security [60], enhances resilience through proactive adaptation to environmental threats [61–63], and underpins flexible coping and community recovery after disruptions [64, 65]. However, place attachment can also lead to risk-blindness, prompting residents to overlook or downplay local hazards like floods or pollution [59, 66]. Research distinguishes between different types of place attachment, which can lead to different outcomes. One important distinction is between a more reflective, self-aware and active attachment to a place, and a more passive form of attachment (e.g. traditional type of attachment) in which places are taken for granted and seen mainly as functional settings or resources for everyday activities (see e.g., Lewicka, [67]. Previous research suggests that active place attachment develops through deliberate exploration and reflexive engagement with one’s surroundings [67], which in turn promotes openness to change [55, 68] and sense of agency in relation to the local environment [69]. It has also been linked to greater trust, broader social capital and involvement in pro-neighbourhood initiatives [67, 70]. In contrast, traditional place attachment is associated with stability-oriented values and a greater threat to the continuity of places, as well as lower openness to newcomers [68] suggesting a reduced capacity for adaptive responses to change. We may thus expect that, while active attachment can foster greater psychological resilience and engagement, traditional attachment may be more closely associated with, for example, heightened threat perceptions, and lower involvement in adaptive responses to crisis-related challenges.

Finally, socioeconomic resources also play a crucial role in shaping individuals’ capacity to maintain or recover well-being amid prolonged crises. Lower socioeconomic status has been consistently associated with heightened vulnerability to mental health difficulties and limited access to coping resources, whereas financial stability and higher education often facilitate adaptive adjustment and upward transitions toward more resilient profiles over time [11, 38]. From a multisystemic perspective, SES operates as both a contextual and structural resource - buffering stress through access to opportunities, security, and social capital, and thereby influencing the likelihood of belonging to, or moving into, more resilient mental-health trajectories. In this sense, SES can be viewed as a structural–contextual resource that expands capacity for other protective processes (e.g., engagement and relationship-building) by reducing chronic strain and increasing access to time and security, thereby indirectly supporting resilient trajectories.

### Current study

In order to capture the complexity of emotional functioning of young adults in times of overlapping social crises, this study employed person-centered analytical methods - LPA and LTA - across three waves of data collection (July 2023, February 2024, and September 2024). By integrating measures of anxiety, depression, life satisfaction, and crisis-related concerns, we aim to identify distinct latent profiles that reflect the diversity of young adults’ emotional responses and well-being. Subsequently, LTA will allow us to track how individuals transition between these profiles over time, revealing patterns of stability, improvement, or deterioration in mental health. Crucially, we will also investigate potential predictors of profile membership and transitions - such as emotion regulation, attachment dimensions, social support, and socioeconomic status - to deepen our understanding of the factors underlying resilience or vulnerability in this population. Drawing on existing research [19, 20], we expected that the most common profile would reflect a resilient pattern - relatively low anxiety and depression, moderate to high life satisfaction, and low to moderate crisis-related concerns - though we anticipated the presence of more distressed and mixed-response profiles as well. We also expected that individuals with greater emotion regulation difficulties and insecure attachment would be more likely to belong to less adaptive profiles, whereas those with higher levels of social support, engagement, active place attachment and place identity, stable socioeconomic conditions would show higher likelihood of resilient profiles. In terms of transitions over time, we expected overall profile stability to be the norm, but with some individuals shifting from less to more adaptive profiles as they adjusted to prolonged and compounding crises. Finally, we hypothesized that risk-related predictors such as emotion dysregulation and insecure attachment would increase the likelihood of deteriorating or persistently maladaptive emotional trajectories, while protective resources would promote movement toward resilient profiles. By identifying nuanced, dynamic profiles and the factors that predict them, this study aims to inform more targeted interventions and design systemic mental-health supports that meet rising needs during overlapping social, economic, and political crises. To this end, while the current study focuses on Poland, this context serves as a unique vantage point for analyzing the psychological dynamics of the global polycrisis. Poland represents a highly relevant case study: as a stable EU and NATO member, it has faced the direct impact of a neighboring land war, a significant refugee influx, and acute economic volatility. In this environment, macro-social threats translate into tangible personal and economic pressures rather than abstract narratives. Consequently, findings from this setting offer critical insights into how mental health trajectories are reshaped when systemic failures become deeply interconnected.

### Situation in Poland during data collection

During the first wave (July 2023), Poland was emerging from the pandemic, absorbing a large influx of Ukrainian refugees, battling inflation, and debating climate risks, AI, and looming national elections. By the second wave (February 2024), a new centrist government had taken office, inflation was easing, refugee support was shifting from emergency relief to long-term integration, and public opinion was increasingly divided between economic anxieties and humanitarian concerns. The third wave (September 2024) unfolded amid renewed economic growth and routine AI use, yet persistent geopolitical tensions at Poland’s eastern border and more frequent extreme weather events kept security and climate on the agenda. Across all three waves, overlapping pressures - post-pandemic adjustment, war-related migration and insecurity, economic volatility, rapid technological change, and intensifying debates over democratic and social rights - created a continuously shifting context for young adults’ mental health and well-being. A detailed description of the situation in Poland during data collection appears in Supplementary Material S1.

## Method

### Participants and procedure

The study was conducted online in three waves using the Ariadna research panel, a Polish internet-based survey platform. Participants were recruited from registered members of the panel through random quota sampling procedures to ensure national representativeness in terms of age, gender, and place of residence. Participants were rewarded with points, which they could exchange for small gifts. For Waves 2 and 3, participants were recontacted through standard panel email reminders inviting them to complete the next survey. Wave 1 conducted in July 2023, included a sample of 1110 Polish adults aged 18 to 30 (M = 24.32, SD = 3.74). Of these, 51.1% identified as women, 48.5% as men, 0.3% as another gender, and 0.2% chose not to disclose. Wave 2, conducted in February 2024, included 434 returning participants (58.5% women, 40.8% men, 0.5% another, 0.2% undisclosed). Wave 3, carried out in September 2024, involved 378 participants (60.1% women, 39.4% men, 0% another, 0.5% undisclosed). Detailed sample characteristics are presented in Supplementary Table S1. This study was approved by the institutional review board of the Faculty of Psychology, University of Warsaw. The presented findings are part of a larger research project on psychological aspects of the overlapping social crises in the Polish young adults.

### Measures

***The Patient Health Questionnaire-9*** (PHQ-9; [71]; Polish version available at www.phqscreeners.com) is a screening instrument designed to assess the risk of depressive disorders including nine items that evaluate the frequency of depressive symptoms over the past two weeks. Items include statements such as ‘Feeling down, depressed, or hopeless’ and ‘Feeling tired or having little energy’. Participants rate each symptom on a scale from 0 (not at all) to 3 (nearly every day), and the scale demonstrated excellent internal consistency in our study (t1: α = 0.897; t2: α = .930, t3: α = 0.932).

***The Generalized Anxiety Disorder-7*** (GAD-7; [72]; Polish version available at www.phqscreeners.com) screens for the risk of generalized anxiety disorder. It consists of seven items that measure the frequency of anxiety-related symptoms during the past two weeks. Items include statements such as ‘Being so restless that it’s hard to sit still’ and ‘Trouble relaxing’. Respondents rate each item on the same 0 (not at all) to 3 (nearly every day) scale, with the measure showing high internal consistency (t1: α = 0.929; t2: α = 0.944, t3: α = 0.946).

***Satisfaction with Life Scale*** ([73]; Polish adaptation: [74]) consists of 5 items. The items were e.g. ‘I am satisfied with my life’ and ‘In most ways my life is close to my ideal’. Answers were given on a scale from 1 - *strongly disagree* to 7 - *strongly agree*; (t1: α = 0.889; t2: α = 0.907, t3: α = 0.923)

***Scale of concerns related to current social crises*** was developed based on instruments from our previous research [8, 9] to measure adolescents’ and young adults’ worries about ongoing social, economic, and environmental issues. An exploratory factor analysis (see Supplementary Material S2 and Supplementary Table S2) identified four distinct subscales: (i) Environmental-Health Concerns (3 items; t1: α = 0.729; t2: α = 0.772, t3: α = 0.792) reflects fears about the climate crisis, ecological disasters, and potential or ongoing pandemic threats; (ii) Socio-Political Concerns (3 items; t1: α = 0.780; t2: α = 0.735, t3: α = 0.771) captures anxieties related to democracy, social rights, and political processes; (iii) Concerns about Artificial Intelligence and Virtual Worlds (3 items; t1: α = 0.779; t2: α = 0.794, t3: α = 0.791) focuses on apprehensions regarding the rapid development of artificial intelligence and the increasing shift of daily activities into virtual or online environments; (iv) Concerns about War and Economic Crisis (5 items; t1: α = 0.777; t2: α = 0.833, t3: α = 0.836) encompasses worries about armed conflict (e.g., the war in Ukraine), broader geopolitical implications, and the risk of worsening economic instability.

***The Difficulties in Emotion Regulation Scale - Short Form*** (DERS-SF; [75]) is a self-report questionnaire assessing various aspects of emotional dysregulation [44]. The DERS-SF comprises 18 items rated on a 5-point scale from 1 (*Almost never*) to 5 (*Almost always*). These items are grouped into six subscales: strategies (e.g. ‘When I’m upset, I believe that there is nothing I can do to make myself feel better’, nonacceptance (e.g. ‘When I’m upset, I feel ashamed with myself for feeling that way’, impulse (e.g. ‘When I’m upset, I become out of control’), goals (e.g. ‘When I’m upset, I have difficulty getting work done’), awareness (e.g. ‘I pay attention to how I feel’), and clarity (e.g. ‘I have difficulty making sense out of my feelings’). The overall score reflects the cumulative difficulties in emotion regulation; however, following the recommendation of Hallion et al., [76], the awareness subscale was excluded from the total score, yielding a Cronbach’s alpha of 0.947.

***The Experiences in Close Relationships-Revised - Short Form*** (ECR-R-SF; [77]; Polish adaptation: [78]) assesses two dimensions of adult attachment: anxiety and avoidance. Attachment anxiety involves fears of rejection, abandonment, or indifference, while attachment avoidance reflects discomfort with intimacy and dependence. Example items include ‘I need a lot of reassurance that I am loved by my partner’ (anxiety) and ‘I get uncomfortable when a romantic partner wants to be very close’ (avoidance). It has 16 items assessed on a 7-point rating from 1 (“I strongly disagree”) to 7 (“I strongly agree”) and maintains strong psychometric properties (α = 0.911 for anxiety, α = 0.873 for avoidance).

***The Social Support Scale*** [5] includes five items that assess the level of social support. It evaluates three dimensions of support [79]: (i) emotional-informational support (e.g. ‘Seeking advice about experienced problems’), (ii) tangible support coupled with positive social interaction (e.g. ‘Taking me to see the doctor when I need it.’), and (iii) affectionate support (e.g. ‘Feeling loved and needed’). Participants indicated the extent of support received on a scale from 1 (definitely not) to 5 (definitely yes), with the measure demonstrating very good internal consistency (α = 0.894).

***The Social Engagement Scale*** is a brief measure designed to assess individuals’ active participation in civic and political activities. It includes three items referring to engagement over the past six months: ‘Signed a petition related to an issue important to me’, ‘Participated in a protest or demonstration’ and ‘Taken part in activities organized by an organization or association focused on social issues’. For each behavior, responses are provided on a 3-point scale: 1 indicates “No, I did not engage in this activity,” 2 means “Yes, I engaged in this activity once,” and 3 signifies “Yes, I engaged in this activity more than once.” (α = 0.676).

***Place Attachment*** was measured with items referred to the village, town or city in which participants live. Participants were asked to respond using a scale from 1 (*strongly disagree*) to 7 (*strongly agree*). *Emotional Place Attachment* was measured with a shortened 3-item scale by Hernandez et al., [80], e.g. ‘I feel attached to my village/town/city’, α = 0.852. *Place Identity* was measured by a 3-item subscale of the shortened place attachment scale by Hernandez et al., [80]; e.g. ‘I identify with this village/town/city’; α = 0.934. *Traditional Place Attachment* was measured with a 3-item scale designed by Lewicka [67]; e.g. ‘Even if there are better places somewhere, I still won’t move from here’, α = 0.831. *Active Place Attachment* was measured with a 3-item scale [67], e.g., ‘I like to keep up with changes in my village/town/city’; α = 0.834.

#### Demographic questions

Participants responded to a series of demographic items included as covariates in the predictor analyses. Perceived financial situation was assessed with the item: “How would you rate your current financial situation compared to other people you know?”, with responses recorded on a 5-point scale ranging from 1 (much worse) to 5 (much better). Size of place of residence was coded on a 5-point ordinal scale, ranging from rural village to a city with over 500,000 inhabitants. Relationship status was assessed with the question: “What is your current relationship status?” with response options: (1) single, (2) in an informal relationship, (3) married, (4) widowed, (5) divorced, (6) separated. For analysis purposes, responses were recoded into a binary variable: 0 = not in a romantic relationship, and 1 = in a committed relationship or married. Education level was recorded based on the highest level of completed education, using an ordinal scale from 1 (primary education) to 7 (postgraduate studies). Past mental health diagnoses were assessed by asking participants to indicate whether they had ever received formal diagnoses from a list of common psychiatric conditions; affirmative responses were summed to yield a total count of self-reported diagnoses.

### Analytical strategy

Analyses were conducted using SPSS (version 29.0) and Mplus (version 8.11; Muthén & Muthén, [81]). To investigate the missing data mechanism, we applied the MissMech procedure in R [82], which provides a robust generalization of Little’s test. Although the non-parametric version indicated no significant departure from a Missing Completely at Random (MCAR) pattern (*p* = .12), subsequent attrition analyses presented in Supplementary Material S4 revealed that participants lost to follow-up reported significantly higher baseline distress (anxiety and depression) than completers. To identify factors independently predicting study retention, we conducted a binary logistic regression. The results showed that attrition was significantly predicted by several sociodemographic and relational factors: age, gender, education, relationship status, and attachment anxiety. Crucially, after controlling for these factors in the multivariate model, neither depressive symptoms (*p* = .069) nor anxiety symptoms (*p* = .569) remained independent predictors of dropout. This pattern suggests that missingness is associated with observed variables already included in our analytical models, which is consistent with the Missing at Random (MAR) assumption. Consequently, we adopted the more conservative Missing at Random (MAR) assumption to ensure the validity of our longitudinal estimates. Missing data were handled using Full Information Maximum Likelihood (FIML), the gold-standard robust approach for Latent Transition Analysis (LTA) under MAR conditions. We explicitly rejected listwise deletion to prevent the systematic bias and loss of statistical power associated with excluding non-completers. To further ensure the robustness of our longitudinal findings, we conducted a sensitivity analysis comparing our primary FIML models with Multiple Imputation (MI) across 20 datasets. The MI-pooled results demonstrated high convergence with FIML estimates across all key parameters, including model fit, classification quality, and transition probabilities (see Supplementary Tables S9 and S10). To ensure constructs remained comparable across the three waves, we performed Multigroup Confirmatory Factor Analysis (MGCFA) to test for longitudinal measurement invariance (configural, metric, and scalar). This procedure utilized the Satorra-Bentler correction. Following the evaluation of invariance using alternative fit indices, we proceeded with person-centered analyses. Then, we determined the number of profiles at Time 1 using LPA. Following recognized guidelines, we employed several criteria to evaluate model fit at each step of the LPA. We examined the Akaike Information Criterion (AIC), Bayesian Information Criterion (BIC), and sample-size adjusted BIC (aBIC), with smaller values indicating better fit. We also employed the entropy index with values ranging between 0 and 1 wherein higher scores denote better model fit. We conducted the Lo–Mendell–Rubin (LMR) and the Bootstrap Likelihood Ratio Test (BLRT) to statistically confirm that adding an extra class significantly improves fit. Finally, we integrated these quantitative indicators with qualitative assessments of theoretical meaningfulness and parsimony, focusing on the size and interpretability of each latent profile. In a second step, we replicated this procedure at Times 2 and 3. Next, we applied LTA, which jointly estimates latent profile structures at multiple time points and models the probability of transition from one profile to another [34]. In this approach, we imposed equality constraints on the intercepts of the measured variables across time, ensuring that the same latent profiles could be meaningfully compared from one wave to the next. Consequently, the proportions of individuals in each profile, as identified by the LTA, sometimes differed slightly from the separate cross-sectional solutions at each time point. Finally, we tested whether emotion regulation difficulties, interpersonal and place attachment, social support and engagement, as well as demographic variables predicted membership in the profiles at Times 1 using three-step multinomial logistic regression. Separately, within the LTA itself, we specified covariate-on‐transition regressions so that the above variables predicted the odds of moving from each profile at wave t to each profile at wave t + 1. Because only five respondents identified as non-binary, these individuals were excluded from this part of the analysis; sex was therefore recoded as female (0) versus male (1) - analytic *N* = 1,105.

## Results

Descriptive statistics and bivariate correlations are presented in Supplementary Table S3.

### Longitudinal measurement invariance

Prior to conducting the LPA/LTA, we tested longitudinal measurement invariance across the three waves using MGCFA (configural, metric, scalar; Satorra–Bentler correction). As presented in Supplementary Table S4, the results supported the assumption of invariance across time. For the majority of constructs, changes in alternative fit indices were well within the strict thresholds proposed by Chen [83] and Zacher and Rudolph [84]. While the War/Economic-Crisis Concerns scale exhibited a slightly larger shift at the scalar level (Delta CFI = 0.020), its overall fit indices remained within acceptable ranges (SRMR = 0.06, RMSEA = 0.09). Given that scalar invariance was fully met for all core mental health indicators, the measurement structure was deemed sufficiently stable for longitudinal comparison.

### Latent profile analysis

Table 1 presents fit indices for two- through six-class solutions across the three waves. We retained the five-class solution as the most parsimonious and substantively interpretable representation of the data across all waves. While information criteria (AIC, BIC, aBIC) and the BLRT continued to improve with additional classes, the Lo–Mendell–Rubin (LMR) test was non-significant for the six-class model in Wave 2 (p = .374) and Wave 3 (p = .310), and the six-class solution yielded a clinically negligible class (n = 3; 0.7%) in Wave 2. Conversely, a four-class solution merged the *Content & Carefree* group with the *Content & Mildly Concerned* group, masking the most resilient pole of the sample. Despite its small size, the *Content & Carefree* profile (approx. 4–6%) emerged consistently in all three waves, supporting its empirical stability. This consistent structure ensured the longitudinal comparability required for the Latent Transition Analysis.

**Table 1:** Fit indices and likelihood-ratio tests for two- through six-class latent-profile models across three waves

Wave (k classes)	Log-likelihood	AIC	BIC	Adj. BIC	Entropy	LMR *p*	BLRT *p*	Class proportions (%)
Wave 1								
2	–14 136.62	28 317.25	28 427.52	28 357.64	0.825	< 0.001	< 0.001	40, 60
3	–13 902.84	27 865.68	28 016.04	27 920.75	0.774	< 0.001	< 0.001	32.3, 30.1, 37.7
4	–13 708.26	27 492.52	27 682.98	27 562.29	0.781	0.001	< 0.001	25.7, 30.3, 15.8, 28.2
5	–13 592.13	27 276.25	27 506.81	27 360.70	0.805	< 0.001	< 0.001	14.5, 26.8, 24.9,30.2, 3.6
6	–13 463.94	27035.88	27306.29	27134.77	0.794	0.038	< 0.001	3.3, 16.6, 30.0, 23.4, 16.4, 9.5
Wave 2								
2	–5 531.61	11 107.22	11 196.83	11 127.01	0.838	-	-	62.2, 37.8
3	–5 418.55	10 897.09	11 019.29	10 924.08	0.838	0.285	< 0.001	10.8, 54.8, 34.3
4	–5 336.01	10 748.01	10 902.79	10 782.19	0.835	0.338	< 0.001	7.4, 47.7, 17.3, 27.6
5	–5 252.65	10 597.30	10 784.66	10 638.68	0.830	0.001	< 0.001	6.5, 22.6, 32.9, 18.4, 19.6
6	–5 174.19	10 456.38	10 675.95	10 504.58	0.858	0.374	< 0.001	7.3, 32.0, 24.2, 0.7, 16.6, 19.2
Wave 3								
2	–4 953.28	9 950.56	10 037.13	9 967.33	0.817	-	-	63.2, 36.8
3	–4 835.57	9 731.14	9 849.19	9 754.01	0.820	0.008	< 0.001	15.3, 55.6, 29.1
4	–4 755.26	9 586.53	9 736.05	9 615.49	0.823	0.100	< 0.001	20.4, 38.9, 29.1, 11.6
5	–4 699.39	9 490.78	9 671.78	9 525.83	0.843	0.359	< 0.001	6.3, 30.2, 37.0, 11.6, 14.8
6	–4 633.14	9 374.28	9 586.47	9 415.14	0.864	0.310	< 0.001	8.0, 2.6, 36.2, 29.4, 9.8, 13.8

**Fig. 1:**
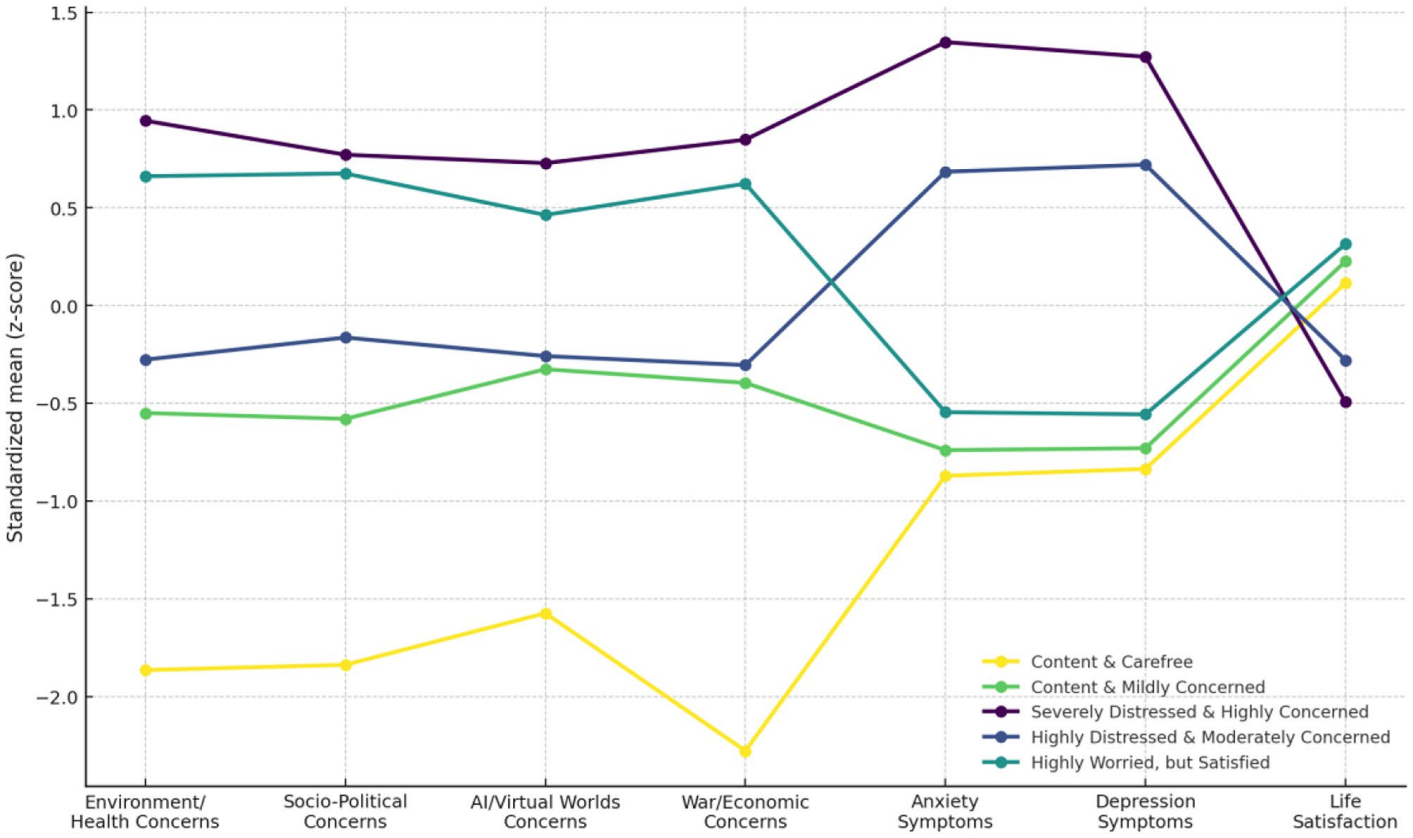
Standardized means for the five latent profiles at Wave 1

The first profile (*n* = 40) was characterized by very low crisis-related concerns, minimal anxiety and depression symptoms, and elevated life satisfaction; it is described as *Content & Carefree*. The second profile (*n* = 335), labeled *Content & Mildly Concerned*, reflects mildly elevated crisis-related concerns, low levels of anxiety and depression, and high life satisfaction. The third profile, *Highly Worried*,* but Satisfied* (*n* = 276), was marked by elevated crisis-related concerns, mildly elevated anxiety and depression symptoms, yet it showed the highest life satisfaction. The fourth profile (*n* = 298), termed *Highly Distressed & Moderately Concerned*, consisted of individuals with moderate crisis-related concerns, high anxiety and depression symptoms, and low life satisfaction. The fifth profile (*n* = 161), labeled *Severely Distressed & Highly Concerned*, represented individuals with very high levels of crisis-related concerns, severe anxiety and depression symptoms, and the lowest life satisfaction. Standardized means for the five profiles at Wave 1 are presented in Fig. 1. Means and standard deviations for all indicators across all three waves are provided in Supplementary Tables S5–S7.

### Latent transition analysis

**Fig. 2:**
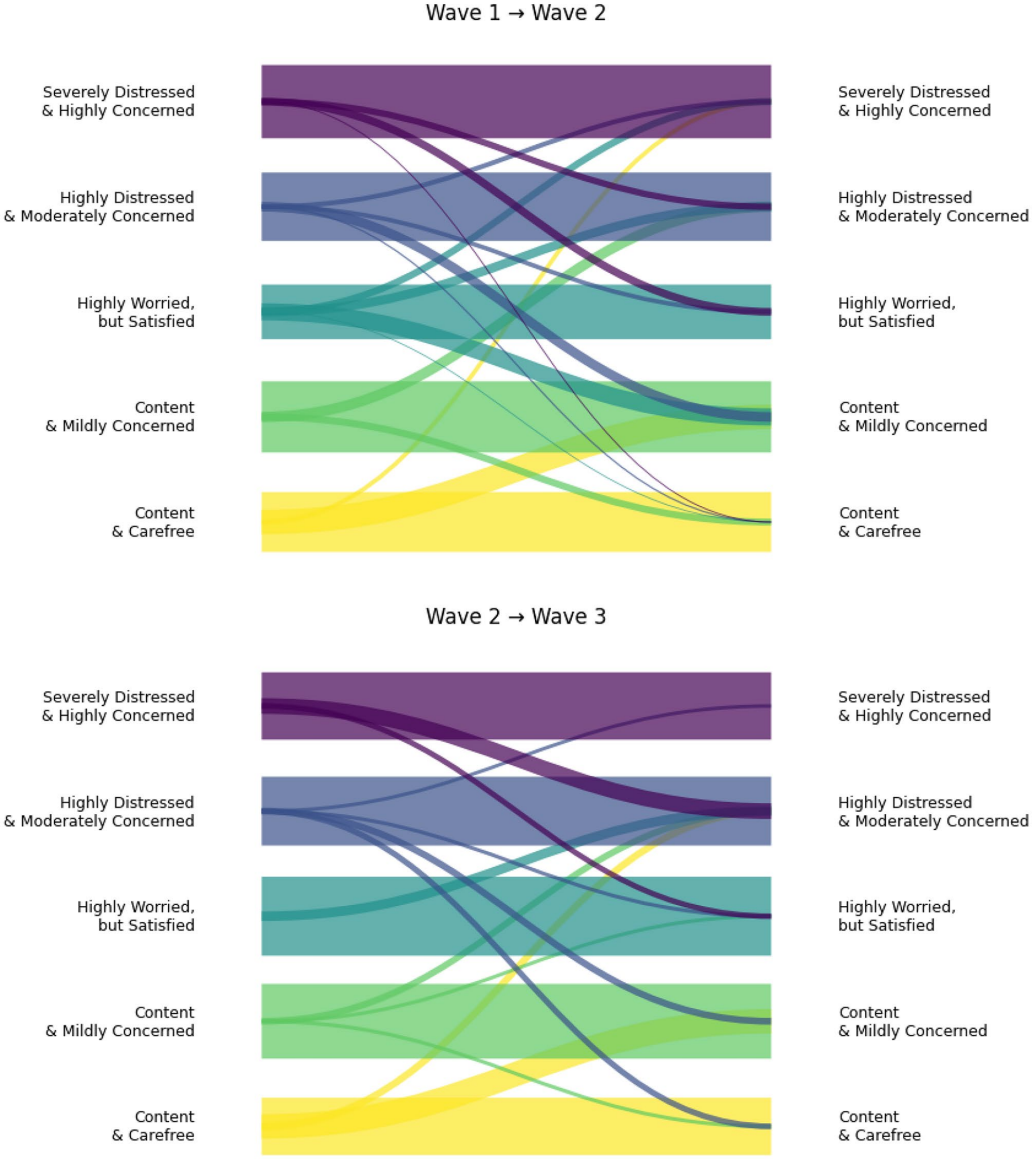
Transitions between first and second wave of the study and between second and third wave of the study. Note. The diagram illustrates the estimated transition probabilities between the five latent profiles from Wave 1 to Wave 2 (top panel) and Wave 2 to Wave 3 (bottom panel). The thickness of the bands corresponds to the probability of transitioning between specific profiles; thicker horizontal bands represent higher profile stability

Next we assessed whether - and how - participants transitioned between these profiles across waves using LTA. Across both intervals, stability (the diagonal) was substantial for most profiles (Fig. 2, Supplementary table S8).

From Wave 1 to Wave 2, profile *Severely Distressed & Highly Concerned* and the *Content & Mildly Concerned* showed the highest stability, followed closely by the *Highly Distressed & Moderately Concerned* class, while the *Content & Carefree* group showed the greatest movement into the *Content & Mildly Concerned* or *Severely Distressed & Highly Concerned* profiles. Between Wave 2 and Wave 3, stability increased further for the *Content & Mildly Concerned* and *Highly Distressed & Moderately Concerned* profiles, and a minority of the *Severely Distressed & Highly Concerned* participants shifted to the *Highly Distressed & Moderately Concerned* and *Highly Worried*,* but Satisfied* class.

### Latent profile and transition predictors

To examine predictors of profile membership at Wave 1, we conducted a three-step multinomial logistic regression with each latent class serving as the reference category. Results of logistic regression with the largest class *Content & Mildly Concerned* as reference category are presented in Table 2, whereas regression analysis for other classes as reference groups are presented in Supplementary Tables S11, S12, S13 and S14.

Several covariates did not significantly discriminate among the profiles and appear to have minimal influence on profile membership. Specifically, participants’ financial situation, age, size of place of residence, education level, romantic relationship status, emotional and traditional place attachment were not significantly associated with any specific class.

In contrast, emotion regulation difficulties emerged as the most robust and consistent predictor of class membership. Participants with greater difficulties in emotion regulation were substantially more likely to belong to the *Severely Distressed & Highly Concerned* profile and the *Highly Distressed & Moderately Concerned* group, relative to other profiles. Conversely, individuals in the *Content & Carefree* and *Content & Mildly Concerned* classes reported significantly lower levels of emotion-regulation difficulties compared to the distressed profiles.

Attachment anxiety also significantly differentiated between profiles. Higher attachment anxiety predicted membership in both distressed profiles (*Severely Distressed & Highly Concerned* and *Highly Distressed & Moderately Concerned*), as well as the *Highly Worried but Satisfied group*. Attachment avoidance, while less consistently predictive, was lower in the *Highly Worried but Satisfied* group compared to the *Highly Distressed & Moderately Concerned* group and *Content & Mildly Concerned* group.

Among place-related predictors, place identity was significantly lower in the S*everely Distressed & Highly Concerned* profile compared to the *Content & Mildly Concerned*, *Highly Worried but Satisfied*, *Highly Distressed & Moderately Concerned*. Active place attachment, in contrast, was significantly higher in the *Highly Worried but Satisfied* profile relative to the *Content & Carefree*, *Content & Mildly Concerned*, and marginally elevated in the *Highly Distressed & Moderately Concerned* group compared to the *Content & Carefree* group.

Gender was a consistent and differentiating factor in profile membership. Men were significantly more likely to belong to the most resilient group - *Content & Carefree* - than to any other profile. They were notably less likely to be classified in the *Severely Distressed & Highly Concerned* and *Highly Worried but Satisfied* profiles. For the *Highly Distressed & Moderately Concerned* profile, the pattern was mixed: men were less likely to be in this group compared to the most resilient profile, but more likely to be in it than in the more severely distressed or worried profiles.

The number of self-reported mental health diagnoses was a meaningful predictor of profile membership. Participants in the *Highly Distressed & Moderately Concerned* profile reported significantly more past diagnoses than those in the *Content & Mildly Concerned* and *Highly Worried but Satisfied* profiles. Similarly, individuals in the *Severely Distressed & Highly Concerned* group had more diagnoses than those in the *Content & Mildly Concerned* profile.

Higher social support significantly predicted membership in the *Highly Worried but Satisfied profile*, distinguishing it from all other profiles. Greater social engagement was associated with increased odds of belonging to the *Highly Worried but Satisfied* profile compared to the *Content & Carefree* and *Content & Mildly Concerned* groups, and to the *Highly Distressed & Moderately Concerned* profile compared to the *Content & Mildly Concerned* profile.

**Table 2:** Multinomial logistic regression predicting Wave-1 latent-profile membership (Reference = Content & Mildly Concerned): Odds Ratios and 95% Confidence Intervals)

Predictor	Content & Carefree OR [95% CI]	Severely Distressed & Highly Concerned OR [95% CI]	Highly Distressed & Moderately Concerned OR [95% CI]	Highly Worried but Satisfied OR [95% CI]
Male gender (0 = female, 1 = male)	4.34 [1.11, 16.92]*	0.24 [0.12, 0.49]***	0.64 [0.38, 1.08]	0.25 [0.15, 0.42]***
Financial situation	0.80 [0.52, 1.23]	0.77 [0.55, 1.07]	0.80 [0.62, 1.02]	0.96 [0.78, 1.19]
Age (years)	1.00 [0.88, 1.14]	1.01 [0.90, 1.12]	0.99 [0.91, 1.08]	1.04 [0.97, 1.12]
Mental-health diagnoses	1.45 [0.67, 3.13]	1.67 [1.08, 2.60]*	1.79 [1.24, 2.59]**	1.21 [0.79, 1.84]
Emotional place attachment	1.07 [0.63, 1.82]	1.37 [0.88, 2.13]	0.94 [0.68, 1.31]	0.99 [0.69, 1.41]
Place identity	1.00 [0.58, 1.73]	0.68 [0.47, 0.99]*	0.98 [0.72, 1.33]	1.09 [0.79, 1.50]
Emotion-regulation difficulties	0.37 [0.14, 0.94]*	22.26 [10.02, 49.45]***	5.58 [3.76, 8.28]***	1.18 [0.77, 1.81]
Attachment anxiety	0.94 [0.66, 1.35]	4.67 [1.79, 12.21]**	1.74 [1.27, 2.37]***	1.48 [1.20, 1.83]***
Attachment avoidance	0.66 [0.38, 1.15]	1.14 [0.50, 2.61]	0.99 [0.66, 1.50]	0.66 [0.50, 0.88]**
Social support	0.97 [0.87, 1.08]	1.08 [0.97, 1.20]	0.99 [0.92, 1.06]	1.12 [1.05, 1.20]***
Social engagement	0.25 [0.03, 2.30]	1.08 [0.49, 2.41]	2.12 [1.21, 3.73]*	2.39 [1.34, 4.24]**
Traditional place attachment	0.66 [0.42, 1.05]	0.96 [0.71, 1.29]	1.00 [0.80, 1.24]	0.90 [0.76, 1.06]
Active place attachment	0.84 [0.59, 1.20]	1.20 [0.87, 1.64]	1.26 [0.98, 1.60]	1.27 [1.05, 1.54]*
Size of place of residence	1.14 [0.83, 1.58]	1.04 [0.79, 1.36]	1.01 [0.85, 1.20]	0.93 [0.78, 1.10]
Level of education	0.88 [0.50, 1.55]	1.31 [0.85, 2.03]	1.08 [0.77, 1.52]	1.05 [0.77, 1.43]
In a romantic relationship	2.53 [0.75, 8.57]	1.78 [0.84, 3.75]	1.15 [0.64, 2.04]	1.11 [0.65, 1.92]

Note. OR = odds ratio; CI = confidence interval. *p < .05; **p < .01; ***p < .001

### Covariates of transitions

Several covariates significantly predicted transitions between profiles (Supplementary Tables S15 and S16). Gender was a predictor of adaptive transitions. From Wave 1 to Wave 2, male participants in the *Highly Distressed & Moderately Concerned* profile had higher odds of transitioning to the *Highly Worried but Satisfied* profile. Conversely, men in the *Highly Worried but Satisfied* profile were significantly less likely to transition into the *Highly Distressed & Moderately Concerned* group. Mental health diagnoses were also predictive, though effects were time-dependent and directionally complex. Between Wave 1 and Wave 2, individuals with more self-reported diagnoses were less likely to transition from the *Highly Distressed & Moderately Concerned* to the *Content & Mildly Concerned* profile and more likely to exhibit the reverse transition. Interestingly, from Wave 2 to Wave 3, higher number of mental health diagnoses predicted positive shifts: individuals in the *Severely Distressed & Highly Concerned*,* Highly Distressed & Moderately Concerned* and *Highly Worried but Satisfied* profile were more likely to move to *Content & Carefree* profile. Attachment avoidance played a significant role: lower avoidance predicted shifts from *Highly Distressed & Moderately Concerned* to *Content & Mildly Concerned*, while higher attachment avoidance increased the odds of deterioration from *Content & Mildly Concerned* to *Highly Distressed & Moderately Concerned* at the first wave. Social support promoted transitions toward the most adaptive profile. From Wave 1 to Wave 2, individuals with higher social support were more likely to shift into the *Content & Carefree* group from nearly all other profiles. Likewise, for those in the *Content & Carefree* profile, social support decreased the odds of moving into the *Severely Distressed & Highly Concerned* or *Highly Distressed & Moderately Concerned* groups. Higher place identity facilitated recovery from Wave 2 to 3, increasing the odds of moving from the *Highly Distressed* profile to the *Content & Mildly Concerned* or *Content & Carefree* profiles. It also helped protect against shifting from the *Content & Carefree* and *Content & Mildly Concerned* profiles into the *Highly Distressed & Moderately Concerned* profile. Importantly, several covariates - including financial situation, age, education, emotional and traditional place attachment, romantic relationship status, and size of place of residence - were not significantly associated with any transitions at either time point, indicating limited influence on longitudinal profile shifts.

## Discussion

The present study adds to the growing body of person-centered research by examining the heterogeneous emotional-response profiles of young adults facing the cumulative pressures of a global polycrisis. In addition, our multisystemic approach - incorporating predictors such as emotion regulation, interpersonal and place attachment, social support, and socioeconomic status - provides a comprehensive view of the factors associated with resilience and various configurations of emotional responses to social crises.

Our person-centered analyses identified five qualitatively distinct subgroups. About 59% of young adults belonged to resilient profiles, characterized by low or mild anxiety and depressive symptoms and high life satisfaction, consistent with previous findings highlighting resilience as the most common response [20, 21]. The largest was the *Content & Mildly Concerned* profile (30.2%) characterized by mild crisis-related worries alongside low anxiety and depression and high life satisfaction, reflecting a largely resilient stance despite ongoing social crises. A small *Content & Carefree* profile (3.6%) showed the lowest levels of crisis worries or emotional symptoms and high well-being. The *Highly Worried but Satisfied* profile (24.9%) exhibited pronounced crisis worries and mild distress but nevertheless high life satisfaction - highlighting that concerns and well-being can coexist, supporting the dual-factor model of mental health ([21, 31–33]). However, the 41% of participants were in two non-resilient profiles (characterized by elevated or severe levels of anxiety and depression symptoms) that substantially exceeds the ~ 30–35% typically observed in prior pandemic and acute-stressor studies (e.g., Bonanno et al., [20], suggesting that prolonged, overlapping crises may further erode coping resources. The *Highly Distressed & Moderately Concerned* group (26.8%) combined moderate crisis concerns with elevated anxiety and depressive symptoms and low well-being. The *Severely Distressed & Highly Concerned* subgroup (14.5%) experienced the most intense worries and symptom severity, coupled with the lowest life satisfaction. These five profiles underscore the heterogeneity of young adults’ responses under compounding social crises. Our results demonstrate that intense crisis-related concerns are not always associated with diminished well-being and can coexist with high life satisfaction. It seems that their effects depend on individuals’ broader regulatory and contextual resources.

Emotion regulation difficulties emerged as the strongest and most consistent risk factor for initial membership in the two profiles characterized by high levels of depressive and anxiety symptoms (both the *Severely Distressed & Highly Concerned* and *Highly Distressed & Moderately Concerned* groups), supporting transdiagnostic frameworks and findings of several studies that emphasize the role of emotion-regulation deficits in development of internalizing psychopathology, especially in times of social crises [8, 41–43]. However, emotion-regulation difficulties did not predict subsequent profile transitions, indicating that once youths are anchored in maladaptive affective states, other resources, particularly social and place‐related attachment factors, such as social support, interpersonal attachment strategies, and place attachment become more salient in driving recovery or further deterioration.

Attachment anxiety and avoidance exhibited distinct, often opposing associations with latent profiles, reflecting their divergent emotion-regulation systems [50]. Higher attachment anxiety predicted membership in distressed profiles, consistent with the use of hyperactivating strategies that amplify threat perception and emotional reactivity [46, 48]. In contrast, attachment avoidance was highest in the *Highly Distressed & Moderately Concerned* group and lowest in the *Highly Worried but Satisfied* class. This aligns with the nature of deactivating strategies: while self-reliance and emotional suppression may initially shield against distress, they ultimately foster isolation and inhibit help-seeking [48, 50]. Consequently, during overlapping crises, the avoidant individual’s detachment appears to limit recovery and social co-regulation opportunities compared to the anxious individual, who - despite greater initial distress - remains emotionally engaged and motivated to seek support.

Longitudinally, lower avoidance facilitated transitions toward the *Content & Mildly Concerned* group, whereas higher avoidance predicted transitions toward highly distressed class. This indicates that avoidant detachment may undermine adaptive adjustment over time by blocking opportunities for flexible coping and interpersonal co-regulation.

Higher perceived social support uniquely distinguished the *Highly Worried but Satisfied* profile from most other groups. Correspondingly, social engagement was highest among the *Highly Worried but Satisfied* group. This subgroup can be interpreted as a phenotype of engaged resilience, in which crisis-related concerns may reflect a rational threat appraisal (functional concern). Within the framework of the Social Identity Model of Collective Action (SIMCA), these concerns may act as a cognitive catalyst; when paired with high social support and engagement, they may translate into purposeful agency rather than being internalized as ruminative distress. In this context, community action may transform concerns into collective purpose, thereby buffering against diminished well-being [55]. Future research using cross-lagged panel models or experimental designs is needed to determine if this engagement actively “buffers” the negative impact of concerns or if high well-being is a prerequisite for such participation. Conversely, the low social engagement observed in the *Severely Distressed & Highly Concerned* group underscores how extreme distress may deplete the motivational and cognitive resources necessary for activism, potentially exacerbating psychological burdens.

Moreover, young adults reporting greater emotional, informational, and tangible support not only maintained higher life satisfaction despite significant crisis-related worries but also showed a greater likelihood of shifting toward the *Content & Carefree* profile from Wave 1 to Wave 2. This pattern suggests that robust support networks may buffer against crisis concerns and facilitate adaptive coping, promoting transitions toward profiles marked by lower distress and elevated well-being.

Active place attachment was highest in the *Highly Worried but Satisfied* and also elevated in the *Highly Distressed & Moderately Concerned* groups. This suggests that active place attachment fosters reflexive awareness, although its role in promoting adaptive outcomes may be context-dependent. While it supports purposeful agency in the *Highly Worried but Satisfied* group, it may also reflect a burdensome recognition of systemic threats in more distressed profiles. This aligns with previous studies indicating that active place attachment facilitates reflective, and potentially adaptive responses to place-related changes [68, 85]. Conversely, low place identity was characteristic of the *Severely Distressed* subgroup, significantly differentiating it from other groups except *Content & Carefree*. Low identification with one’s environment may thus intensify psychological distress or reflect indifference toward crises. At the same time, in the long run, place identity appears to protect against psychological strain, as it predicted transitions to low-distress classes. Passive forms of place attachment, such as traditional attachment (long-term familiarity), did not significantly predict group membership or transitions, indicating that mere habitual rootedness does not sufficiently buffer stress.

These findings highlight that active, reflective attachment to place - characterized by agency, emotional investment, and symbolic meaning - supports psychological stability and resilience during crises, consistent with broader environmental psychology research [59, 86]. In this sense, we can also refer to a *place cure* effect whereby place attachment serves as a resource that helps individuals cope with crisis-related disruptions [87].

Overall, the *Highly Worried but Satisfied* subgroup was characterized by a combination of elevated social support, social engagement, and active place attachment along with slightly elevated emotion-regulation difficulties and attachment anxiety. This profile suggests that robust social ties combined with emotional sensitivity may be associated with higher engagement and resilience despite increased crisis-related worries. It underscores the multidimensional nature of resilience, which emerges not only from personal regulatory capacities but also through collective engagement, fostering shared agency and social cohesion.

Men were overrepresented in the two profiles characterized by the lowest anxiety and depression symptoms and underrepresented in the *Severely Distressed & Highly Concerned* group, and were less likely to move to the highly distressed profile over time, aligning with previous studies on gender differences in internalizing symptoms (e.g. Platt et al., [3], Thapar, [88]. Mental health diagnoses showed complex effects: having more diagnoses predicted persistence of distress between Waves 1 and 2, yet also increased the likelihood of shifting from severe distress into milder profiles by Wave 3 - perhaps because a formal diagnosis may foster greater symptom awareness and sustained treatment engagement over time.

Between Waves 1 and 2, the subgroup with the highest distress and concerns remained remarkably stable, reflecting compounded vulnerabilities - emotion-regulation difficulties, elevated attachment anxiety, and weak place identity - that likely perpetuated their cycle of distress. However, stability decreased from Wave 2 to Wave 3 as some individuals transitioned into more adaptive profiles (*Content & Mildly Concerned* or *Content & Carefree*). This suggests that even severe distress can improve over time, possibly due to enhanced access to coping resources, social support, or societal adaptations to ongoing crises. Conversely, the *Content & Mildly Concerned* group showed the highest stability from Wave 2 to Wave 3, likely indicating that as social crises persisted, these young adults adapted or became accustomed to their circumstances, settling into a stable equilibrium characterized by modest concerns, low levels of distress, and sustained high life satisfaction.

### Limitations and future directions

Despite yielding valuable insights, the current study has several limitations. First, our online panel sample may limit generalizability. Second, exclusive reliance on self-report measures increases the risk of common method variance (CMV) and social desirability bias. CMV may have inflated associations among study variables (e.g., internalizing symptoms and emotion regulation difficulties), potentially contributing to clearer separation of latent profiles and more stable transition patterns than might be observed with multi-method data; social desirability may also have led to underreported distress or overreported engagement, biasing profile proportions toward more resilient groups. Third, the observational design precludes causal inference. Although we modeled demographic, individual, and relational resources as predictors of profile membership, reciprocal processes are plausible: higher well-being and lower distress may facilitate engagement and perceptions of support, whereas sustained crisis exposure and chronic distress may undermine regulatory capacity and reduce engagement with supportive contexts. Relatedly, some predictors (e.g., attachment strategies and perceived social support) may themselves change in response to prolonged instability, and unmeasured stable characteristics (e.g., personality traits such as neuroticism/extraversion) may contribute to both perceived resources and symptom reporting. Future research using cross-lagged or within-person longitudinal models and multi-method assessments is needed to clarify directionality and mechanisms. Fourth, some predictors showed modest internal consistency (e.g., the brief Social Engagement scale, α = 0.676), and some measures were relatively brief or adapted (including the locally developed crisis-related concerns scales). While longitudinal invariance testing generally supported construct stability, War/Economic-Crisis Concerns showed minor residual non-invariance at the scalar level; accordingly, transitions characterized primarily by changes in this concern domain should be interpreted with additional caution. Fifth, our analytical approach required several trade-offs regarding demographic representation. Due to low frequency, we were unable to include non-binary participants or analyze relationship status in high detail, limiting insight into intersectional vulnerabilities. Sixth, attrition across waves was relatively high and selective with respect to baseline internalizing symptoms, raising the possibility of a “healthy survivor” bias despite the use of FIML and sensitivity checks. Finally, the Polish context - particularly its geopolitical proximity to the war in Ukraine - may limit generalizability to more stable or differently volatile regions. Replication across other age cohorts, cultural contexts, and socioeconomic groups, oversampling of minority groups, stronger retention strategies, and mixed-method approaches would help strengthen future evidence and enrich understanding of lived experiences underlying transitions. Additionally, subsequent studies could explore further individual and contextual predictors of resilience, such as mentalizing abilities, empathy, and sense of community.

### Practical implications

From a practical standpoint, our findings highlight several priority targets that may plausibly support adaptive functioning, while recognizing that these implications are based on observational, self-reported associations and therefore require rigorous evaluation in applied settings. Emotion regulation emerged as the most robust individual-level correlate: greater difficulties consistently distinguished the highly distressed profiles, suggesting that strengthening emotion-regulation skills may be a promising focus for prevention and intervention efforts. Relational and contextual resources - particularly social support, social engagement, and active place attachment - were characteristic of individuals who either remained within or transitioned toward less distressed profiles. These resources were especially central to the *Highly Worried but Satisfied* profile, supporting the hypothesis that crisis-related concerns can co-occur with preserved well-being when young adults are embedded in supportive community contexts. Accordingly, interventions that strengthen supportive peer networks and create opportunities for meaningful engagement may represent logical targets for promoting adjustment during overlapping crises. Such collective processes may be particularly important because shared group norms can foster solidarity and collective efficacy, motivating adaptive behaviors in challenging contexts [89].

At the same time, the low engagement observed among the most distressed participants suggests that intervention effects may be non-linear. Intensive clinical support may be needed first to restore the motivational and cognitive resources required to benefit from community-level programs. Rather than a single linear strategy, our findings point to a tiered multisystemic approach that combines individual-level clinical stabilization with opportunities for relational and community engagement. Future intervention and translational research, including randomized and pragmatic trials should test the effectiveness and feasibility of these components, both independently and in combination, in a polycrisis context.

## Supplementary Information

Below is the link to the electronic supplementary material.

Supplementary Material 1

## Data Availability

The dataset from the current study is available in the Open Science Framework (OSF) repository: [https://osf.io/yd9nj/].
