# Supplementary material for: Young adults in times of overlapping social crises: dynamic profiles of mental health and crisis-related concerns using latent transition analysis
Source: Global Health. 2026 Feb 27;22:35. doi: 10.1186/s12992-026-01199-8 (PMC13049869; doi:10.1186/s12992-026-01199-8)
Supplement: Supplementary file 1 — Supplementary Material 1 [file 12992_2026_1199_MOESM1_ESM.docx]

**Supplementary Material (SM)**

**S1. Detailed description of situation in Poland during data collection**

**Wave 1: July 2023**COVID-19 was no longer dominating daily life and the epidemic emergency ended  (Furlong, 2023). Over a million Ukrainian refugees had poured into Poland after Russia’s full-scale invasion in 2022, and by July 2023 an estimated 1.8 million war refugees were still living in Poland (Korzeniewski​ et al, 2024).  This massive influx – mostly women and children – put strain on housing, schools, and social services, even as Poles continued to show solidarity. The government, facing a prolonged refugee presence, began shifting from emergency aid to long-term measures. In July 2023, Poland was grappling with high inflation and economic uncertainty. Inflation remained high (10–11%) but was lower than earlier peaks (Monetary Policy Council, 2023). The cost-of-living crisis dominated headlines, with food and housing affordability a key concern. Summer 2023 brought extreme weather to Europe, and Poland was no exception. A July heatwave pushed temperatures in parts of Poland above 35°C​, underscoring the growing impact of climate change. A global artificial intelligence boom was underway in 2023, and Polish society was actively engaging with the trend. The release of tools like OpenAI’s ChatGPT had sparked widespread discussion by mid-2023. Debates focused on education and regulation, with attention to the EU AI Act and the need for policies on generative AI (Yaros et al., 2023). Politically, July 2023 marked the beginning of the parliamentary campaign season ahead of national elections scheduled for October. Public discourse was dominated by issues such as the economy, security, migration, and rule of law. Notably, women’s rights remained a polarizing topic in the campaign. Controversial laws introduced in earlier years—such as the near-total abortion ban—were still in force and drew protests and criticism from national and international observers. Women’s rights organizations were active in raising awareness and supporting those affected, while opposition parties promised to reverse or revise the restrictive reproductive laws if elected. Public debate also focused on tensions along the Poland-Belarus border. During this time the Polish government reported increasing incidents involving groups of migrants directed toward the frontier by Belarusian authorities. These border provocations reinforced public anxiety about national security and the potential for regional escalation (Ministry of the Interior and Administration, 2023).

**Wave 2: February 2024**Following the October 2023 elections, a new centrist government led by Donald Tusk came to power. The administration focused on restoring ties with the EU, fiscal discipline, and refugee integration. COVID-19 faded into the background. In Poland, the initial refugee emergency had evolved into a long-term reality. The number of Ukrainian refugees in Poland had dropped and stabilized at just under one million by early 2024 as some refugees returned home or moved to other countries (Toth, 2024)​. However, public sentiment was more mixed than at the start of the war – while most Poles remained supportive, polling showed support for hosting Ukrainians had fallen from 94% in March 2022 to around 65% by late 2023​. This reflected a degree of “compassion fatigue” and domestic political rhetoric capitalizing on economic anxieties. Inflation dropped sharply (to 2–3%). Consumer sentiment improved. GDP growth was expected to resume (European Commission, Directorate-General for Economic and Financial Affairs. 2024). Youth activism and media attention kept climate on the public agenda and AI discourse continued. The migrant crisis on the Polish-Belarusian border continues. Polish government made public that in the last 6 months, 6000 migrants and asylum seekers attempted to cross the Belarusian border (Ptak, 2024). Public opinion in Poland remained divided: while some segments of the population, particularly in border regions, express fear and resistance toward the presence of migrants—often citing security concerns—others criticize the state's approach as inhumane and argue that it constitutes a violation of fundamental human rights.

**Wave 3: September 2024**The war in Ukraine continued with little change on the front. Refugees became more integrated into Polish society. Economic growth returned, inflation stabilized, and real wages rose. Consumer confidence was cautiously optimistic. A record-breaking summer heatwave and Vistula River drought dominated headlines, followed by flash floods in parts of southern Poland, highlighting climate instability. Demands for water infrastructure and climate adaptation increased. Calls for climate action intensified. The use of AI sparked public debate and became part of daily life and political discourse. On the political and social front, women’s rights continued to feature in national debate. Debates intensified around reintroducing legal abortion —a reversal of the 2020 restrictive Constitutional Tribunal ruling, as well as around sex education in schools and reproductive health services, prompting mobilization both by feminist organizations and conservative groups. Tension around the migrant crisis escalated as in mid-2024 the Polish government reinstated a 60 kilometer “exclusion zone” along parts of the eastern frontier, significantly increased its military presence, and tightened border controls. In September 2024 the exclusion zone was extended for three more months (Rankin, 2025). These developments, alongside the ongoing war in Ukraine and regular NATO exercises, contributed to a broader sense of geopolitical insecurity in Poland.

**References**

European Commission, Directorate-General for Economic and Financial Affairs. (2024, November 15). *Economic forecast for Poland*.<https://economy-finance.ec.europa.eu/economic-surveillance-eu-economies/poland/economic-forecast-poland_en>

Furlong, A. (2023, May 5). WHO declares end of COVID-19 global health emergency. *Politico Pro*. https://www.politico.eu/article/world-health-organization-end-coronavirus-global-emergency/

Korzeniewski, K., Shkilna, M., Huk, M., Shevchuk, O., & Marchelek-Myśliwiec, M. (2024). Ukrainian war refugees and migrants in Poland: implications for public health. *Journal of Travel Medicine*, *31*(1), taad119.

Ministry of the Interior and Administration. (2023, August 30). *Briefing on the situation on the border with Belarus with Minister Mariusz Kamiński*.<https://www.gov.pl/web/mswia-en/briefing-on-the-situation-on-the-border-with-belarus-with-minister-mariusz-kaminski>

Monetary Policy Council. (2023, July). *Inflation report – July 2023*. Narodowy Bank Polski. https://nbp.pl/wp-content/uploads/2023/07/Inflation-Report-%E2%80%93-July-2023.pdf

Ptak, A. (2024, February 7). *Poland publishes data on thousands of migrant “pushbacks” at Belarus border for first time*. Notes From Poland.<https://notesfrompoland.com/2024/02/07/poland-publishes-data-on-thousands-of-migrant-pushbacks-at-belarus-border-for-first-time/>

Rankin, J. (2025, February 4). *‘They are people’: Asylum seekers caught up in ‘hybrid war’ at Poland–Belarus border*. The Guardian.<https://www.theguardian.com/world/2025/feb/04/they-are-people-asylum-seekers-caught-up-hybrid-war-poland-belarus-border>

Toth, K. (2024, March 5). *After two years in Poland, Ukrainian refugees ask when – and if – they will go home*. The New Humanitarian. <https://www.thenewhumanitarian.org/news-feature/2024/03/05/poland-ukrainian-refugees-ask-when-they-will-go-home>

Yaros, O., Bruder, A. H., & Peters, S. (2023, June 16). *European parliament reaches agreement on its version of the proposed EU artificial intelligence act*. Mayer Brown.<https://www.mayerbrown.com/en/insights/publications/2023/06/european-parliament-reaches-agreement-on-its-version-of-the-proposed--eu-artificial-intelligence-act>

**Table S1**

*Description of the participants in three waves.*

| Variables | | Wave 1  (N = 1110) | | Wave 2  (N = 434) | | Wave 3  (N = 378) | |
| --- | --- | --- | --- | --- | --- | --- | --- |
|  |  | N | Percent | N | Percent | N | Percent |
| Age |  |  |  |  |  |  |  |
|  | 18-24 | 563 | 50.7 | 171 | 39.4 | 140 | 37.0 |
|  | 25-34 | 547 | 49.3 | 263 | 60.6 | 238 | 63.0 |
| Gender |  |  |  |  |  |  |  |
|  | Female | 567 | 51.1 | 254 | 58.5 | 227 | 60.1 |
|  | Male | 538 | 48.5 | 177 | 40.8 | 149 | 39.4 |
|  | Other | 3 | 0.3 | 1 | 0.2 | 0 | 0 |
|  | I don’t want to answer this question | 2 | 0.2 | 2 | 0.5 | 2 | 0.5 |
| Residence |  |  |  |  |  |  |  |
|  | Village | 428 | 38.6 | 170 | 39.2 | 143 | 37.8 |
|  | Small town (up to 20,000 residents | 153 | 13.8 | 59 | 13.6 | 41 | 10.8 |
|  | Medium town (20,000-99,000 residents) | 211 | 19.0 | 73 | 16.8 | 69 | 18.3 |
|  | Big town (100,000-500,000 residents) | 196 | 17.7 | 83 | 19.1 | 83 | 22.0 |
|  | Big city (over 500,000 residents) | 122 | 11.0 | 49 | 11.3 | 42 | 11.1 |
| Education |  |  |  |  |  |  |  |
|  | Primary or secondary school education | 53 | 4.8 | 9 | 2.1 | 7 | 1.9 |
|  | Basic vocational, secondary vocational, or post-secondary | 90 | 8.1 | 39 | 9.0 | 29 | 7.7 |
|  | Secondary education | 424 | 38.2 | 150 | 34.6 | 122 | 32.3 |
|  | Post-secondary education | 153 | 13.8 | 45 | 10.4 | 44 | 11.6 |
|  | Higher education - Bachelor | 137 | 12.3 | 58 | 13.4 | 51 | 13.5 |
|  | Higher education - Master | 222 | 20.0 | 115 | 26.5 | 109 | 28.8 |
|  | Higher education - PhD and above | 31 | 2.8 | 18 | 4.1 | 16 | 4.2 |
| Material situation |  |  |  |  |  |  |  |
|  | Well below average | 63 | 5.7 | 29 | 6.7 | 25 | 6.6 |
|  | Below average | 91 | 8.2 | 35 | 8.1 | 29 | 7.7 |
|  | Rather below average | 128 | 11.5 | 50 | 11.5 | 47 | 12.4 |
|  | Average | 515 | 46.4 | 204 | 47.0 | 178 | 47.1 |
|  | Rather above average | 223 | 20.1 | 84 | 19.4 | 69 | 18.3 |
|  | Above average | 72 | 6.5 | 25 | 5.8 | 24 | 6.3 |
|  | Well above average | 18 | 1.6 | 7 | 1.6 | 6 | 1.6 |
| Marital status |  |  |  |  |  |  |  |
|  | Single | 470 | 42.3 | 176 | 40.6 | 155 | 41.0 |
|  | In an informal relationship | 410 | 36.9 | 157 | 36.2 | 138 | 36.5 |
|  | In a formal relationship | 218 | 19.6 | 98 | 22.6 | 82 | 21.7 |
|  | Widow/er | 3 | 0.3 | 1 | 0.2 | 1 | 0.3 |
|  | Divorced | 6 | 0.5 | 2 | 0.5 | 2 | 0.5 |
|  | Separated | 3 | 0.3 | 0 | 0 | 0 | 0 |

**S2. Factor Analysis for the Scale of concerns related to current social crises**

Before conducting the factor analysis, we verified that the data met the necessary assumptions. The Kaiser–Meyer–Olkin (KMO) measure was .876, indicating very good sampling adequacy. Bartlett’s Test of Sphericity was also significant, χ²(105) = 6289.33, p < .001, confirming that the correlation matrix was factorable. An exploratory factor analysis (EFA) was performed using an oblique (oblimin) rotation to assess correlations among the factors. The eigenvalues and the scree plot supported a four-factor solution, with each factor’s eigenvalue exceeding 1.0. The four retained factors accounted for 63.40% of the total variance. Factor 1 (eigenvalue = 5.509) explained 36.73% of the variance, Factor 2 (eigenvalue = 1.630) explained 10.86%, Factor 3 (eigenvalue = 1.353) explained 9.02%, and Factor 4 (eigenvalue = 1.019) explained 6.80%. The pattern matrix shows the final loadings for the four-factor solution. The factors can be summarized as follows: (i) Socio-Political and Mental Health Concerns (4 items). Items address threats to democracy, women’s rights, election outcomes, and the adequacy of mental health care. This factor highlights worries about political stability, civil rights, and mental health services. (ii) AI and Virtual World Concerns (3 items). Items focus on the rapid development of artificial intelligence, the shift of daily activities into virtual spaces, and potential job market disruptions. This factor captures apprehensions regarding technological advances and their societal impacts. (iii) War and Economic Crisis Concerns (5 items). Items relate to the war in Ukraine’s effect on Poland’s economy, the influx of refugees, deepening financial or energy crises, and the possibility of the war spreading. This factor reflects fears of armed conflict, geopolitical instability, and economic repercussions. (iv) Climate and Epidemic Concerns (3 items). Items involve the immediate and long-term effects of the climate crisis, such as extreme weather events, and the risk of future epidemics. This factor centers on environmental threats and public health emergencies. One of the items (Results of the parliamentary elections in Poland) was removed in the phase of further waves from Socio-Political and Mental Health Concerns (4 items) since the elections were over, so the final version of this subscale included 3 items.

**Table S2**

*Exploratory factor analysis*

|  | Component 1 | Component 2 | Component 3 | Component 4 |
| --- | --- | --- | --- | --- |
| Threats to democracy in Poland | **.782** |  |  |  |
| Results of the parliamentary elections in Poland | **.769** |  |  |  |
| Threats to women's rights in Poland | **.741** |  |  |  |
| Lack of adequate mental health care in Poland | **.605** |  |  |  |
| Challenges related to the development of artificial intelligence, including ChatGPT |  | **.899** |  |  |
| Challenges related to the increasing shift of activities to virtual worlds and the internet |  | **.817** |  |  |
| Dramatic changes in the labor market (e.g., mass layoffs) caused by the development of artificial intelligence |  | **.729** |  |  |
| The impact of the war in Ukraine on the Polish economy |  |  | **-.800** |  |
| Challenges related to the presence of a large number of refugees from Ukraine in Poland |  |  | **-.736** |  |
| The risk of an escalating economic and financial crisis in Poland and worldwide | .328 |  | **-.578** |  |
| The risk of a deepening energy crisis, including disruptions in the supply of gas or electricity in Poland |  |  | **-.577** |  |
| The risk of the war in Ukraine spreading to Polish territory |  |  | **-.549** | -.364 |
| Current consequences of the climate crisis (e.g., an increase in violent storms, higher temperatures, weather anomalies, food shortages) |  |  |  | **-.737** |
| Threats related to the climate crisis that will affect future generations |  |  |  | **-.734** |
| The risk of further epidemics |  |  |  | **-.605** |

**Confirmatory Factor Analysis (Waves 2 and 3)**

To validate the factor structure identified in the Wave 1 by EFA, we conducted Confirmatory Factor Analyses (CFA) using data from Wave 2 and Wave 3.

Wave 2 Findings

The four-factor model was specified based on the Wave 1 EFA structure. Examination of modification indices suggested that model fit could be improved by allowing error terms for two pairs of items to correlate. The resulting measurement model demonstrated good fit to the data: χ2(69) = 165.84, p < .001; CFI = 0.943; SRMR = 0.046; RMSEA = 0.057. All standardized factor loadings were statistically significant (p < .001) and robust, ranging from .617 to .783.

Wave 3 Findings

We replicated the CFA using data from Wave 3. Consistent with the Wave 2 model, two pairs of error terms were allowed to correlate to address modification indices. The Wave 3 model also yielded acceptable fit indices: χ2(69) = 178.74, p < .001; CFI = 0.937; SRMR = 0.061; RMSEA = 0.065. All factor loadings were statistically significant (p < .001), ranging from .611 to .896.

**Table S3a**

*Descriptive statistics and within-construct correlations across waves*

|  |  | M | SD | 1 | 2 | 3 | 4 | 5 | 6 | 7 | 8 | 9 | 10 | 11 | 12 | 13 | 14 | 15 | 16 | 17 | 18 | 19 | 20 |
| --- | --- | --- | --- | --- | --- | --- | --- | --- | --- | --- | --- | --- | --- | --- | --- | --- | --- | --- | --- | --- | --- | --- | --- |
| Environmental-Health Concerns – Wave 1 | 1 | 3.37 | 0.91 |  |  |  |  |  |  |  |  |  |  |  |  |  |  |  |  |  |  |  |  |
| Environmental-Health Concerns – Wave 2 | 2 | 3.20 | 0.94 | .56^***^ |  |  |  |  |  |  |  |  |  |  |  |  |  |  |  |  |  |  |  |
| Environmental-Health Concerns – Wave 3 | 3 | 3.20 | 0.95 | .58^***^ | .69^***^ |  |  |  |  |  |  |  |  |  |  |  |  |  |  |  |  |  |  |
| Socio-Political Concerns – Wave 1 | 4 | 3.71 | 0.98 | .54^***^ | .40^***^ | .40^***^ |  |  |  |  |  |  |  |  |  |  |  |  |  |  |  |  |  |
| Socio-Political Concerns – Wave 2 | 5 | 3.46 | 0.93 | .44^***^ | .57^***^ | .42^***^ | .62^***^ |  |  |  |  |  |  |  |  |  |  |  |  |  |  |  |  |
| Socio-Political Concerns – Wave 3 | 6 | 3.40 | 0.97 | .47^***^ | .50^***^ | .59^***^ | .61^***^ | .61^***^ |  |  |  |  |  |  |  |  |  |  |  |  |  |  |  |
| Concerns about Artificial Intelligence and Virtual Worlds – Wave 1 | 7 | 3.27 | 0.92 | .41^***^ | .30^***^ | .34^***^ | .31^***^ | .32^***^ | .34^***^ |  |  |  |  |  |  |  |  |  |  |  |  |  |  |
| Concerns about Artificial Intelligence and Virtual Worlds – Wave 2 | 8 | 3.18 | 0.89 | .32*** | .49^***^ | .41^***^ | .26^***^ | .45^***^ | .41^***^ | .43^***^ |  |  |  |  |  |  |  |  |  |  |  |  |  |
| Concerns about Artificial Intelligence and Virtual Worlds – Wave 3 | 9 | 3.17 | 1.02 | .28^***^ | .42^***^ | 0.53^***^ | .21^***^ | .27^***^ | .44^***^ | .36^***^ | .46^***^ |  |  |  |  |  |  |  |  |  |  |  |  |
| Concerns about War and Economic Crisis – Wave 1 | 10 | 3.58 | 0.77 | .50^***^ | .33^***^ | .33^***^ | .44^***^ | .38^***^ | .36^***^ | .39^***^ | .30^***^ | .27^***^ |  |  |  |  |  |  |  |  |  |  |  |
| Concerns about War and Economic Crisis – Wave 2 | 11 | 3.51 | 0.82 | .38^***^ | .56^***^ | .41^***^ | .34^***^ | .52^***^ | .38^***^ | .31^***^ | .53^***^ | .39^***^ | .56^***^ |  |  |  |  |  |  |  |  |  |  |
| Concerns about War and Economic Crisis – Wave 3 | 12 | 3.48 | 0.83 | .38^***^ | .46^***^ | .49^***^ | .29^***^ | .39^***^ | .47^***^ | .33^***^ | .46^***^ | .56^***^ | .52^***^ | .63^***^ |  |  |  |  |  |  |  |  |  |
| Symptoms of depression – Wave 1 | 13 | 9.18 | 6.36 | .19^***^ | .23^***^ | .23^***^ | .19^***^ | .22^***^ | .26^***^ | .13^***^ | .16^**^ | .23^***^ | .13^***^ | .20^***^ | .19^***^ |  |  |  |  |  |  |  |  |
| Symptoms of depression – Wave 2 | 14 | 8.88 | 6.79 | .21^***^ | .27^***^ | .25^***^ | .16^***^ | .24^***^ | .23^***^ | .14^**^ | .26^***^ | .19^**^ | .16^***^ | .26^***^ | .26^***^ | .66^***^ |  |  |  |  |  |  |  |
| Symptoms of depression – Wave 3 | 15 | 8.95 | 7.10 | .19^***^ | .26^***^ | .24^***^ | .18^***^ | .17^**^ | .22^***^ | .23^***^ | .23^***^ | .26^***^ | .14^**^ | .26^***^ | .25^***^ | .62^***^ | .71^***^ |  |  |  |  |  |  |
| Symptoms of anxiety – Wave 1 | 16 | 7.51 | 5.62 | .22^***^ | .25^***^ | .26^***^ | .20^***^ | .23^***^ | .28^***^ | .18^***^ | .21^***^ | .25^***^ | .15^***^ | .19^***^ | .20^***^ | .81^***^ | .61^***^ | .59^***^ |  |  |  |  |  |
| Symptoms of anxiety – Wave 2 | 17 | 7.41 | 5.79 | .26^***^ | .30^***^ | .26^***^ | .22^***^ | .29^***^ | .25^***^ | .17^***^ | .27^***^ | .21^***^ | .17^***^ | .27^***^ | .29^***^ | .60^***^ | .83^***^ | .65^***^ | .64^***^ |  |  |  |  |
| Symptoms of anxiety – Wave 3 | 18 | 7.57 | 5.93 | .27^***^ | .29^***^ | .26^***^ | .23^***^ | .21^***^ | .27^***^ | .27^***^ | .25^***^ | .30^***^ | .21^***^ | .28^***^ | .28^***^ | .55^***^ | .65^***^ | .87^***^ | .59^***^ | .67^***^ |  |  |  |
| Life satisfaction – Wave 1 | 19 | 19.66 | 6.23 | .01 | .10^*^ | .02 | -.07^*^ | .00 | -.06 | .03 | .02 | -.08 | -.01 | .00 | -.02 | -.36^***^ | -.23^***^ | -.32^***^ | -.29^***^ | -.21^***^ | -.26^***^ |  |  |
| Life satisfaction – Wave 2 | 20 | 20.07 | 6.18 | .04 | .10^*^ | .03 | -.01 | .08 | .02 | .08 | .08 | -.02 | .01 | .04 | -.04 | -.31^***^ | -.28^***^ | -.34^***^ | -.23^***^ | -.23^***^ | -.30^***^ | .69^***^ |  |
| Life satisfaction – Wave 3 | 21 | 20.11 | 6.62 | .03 | .11 | .06 | -0.05 | .13* | .04 | .01 | .06 | -0.01 | .02 | .04 | -.01 | -.33^***^ | -.30^***^ | -.34^***^ | -.25^***^ | -.26^***^ | -.25^***^ | .65^***^ | .70^***^ |

**Table S3a**

*Stable Predictors and Covariates*

|  |  | M | SD | 1 | 2 | 3 | 4 | 5 | 6 | 7 | 8 | 9 | 10 | 11 |
| --- | --- | --- | --- | --- | --- | --- | --- | --- | --- | --- | --- | --- | --- | --- |
| Attachment avoidance | 1 | 3.27 | 1.11 |  |  |  |  |  |  |  |  |  |  |  |
| Attachment anxiety | 2 | 4.13 | 1.33 | -.06^*^ |  |  |  |  |  |  |  |  |  |  |
| Social Support | 3 | 21.55 | 5.33 | -.57^***^ | -.20^***^ |  |  |  |  |  |  |  |  |  |
| Place identity | 4 | 4.21 | 1.66 | -.15^***^ | .02 | .19^***^ |  |  |  |  |  |  |  |  |
| Social engagement | 5 | 1.42 | 0.52 | -.00 | .12^***^ | .02 | .01 |  |  |  |  |  |  |  |
| Difficulties in emotion regulation | 6 | 2.47 | 0.90 | .13^***^ | .42^***^ | -.21^***^ | -.06^*^ | .22^***^ |  |  |  |  |  |  |
| Emotional attachment to place | 7 | 4.43 | 1.53 | -.16^***^ | .00 | .24^***^ | .80^***^ | -.02 | -.08* |  |  |  |  |  |
| Traditional place attachment | 8 | 10.82 | 4.74 | .03 | .01 | .02 | .48^***^ | -0.01 | .04 | .50^***^ |  |  |  |  |
| Active place attachment | 9 | 12.57 | 4.36 | -0.16^***^ | .03 | .20^***^ | .51^***^ | .08^**^ | -0.03 | .52^***^ | .39^***^ |  |  |  |
| Diagnosis | 10 | 0.56 | 1.05 | -0.02 | .21^***^ | -0.07^*^ | -0.07^*^ | .25^***^ | .29^***^ | -0.05 | -0.08^**^ | .06 |  |  |
| Being in a relationship | 11 | 0.57 | .50 | -.26^***^ | -.07^*^ | .26^***^ | -.05 | .03 | -.04 | -.05 | -.07^*^ | .05 | .00 |  |
| Socioeconomic status | 12 | 1.81 | .83 | -.21^***^ | -.09^**^ | .21^***^ | -.02 | .01 | -.06 | -.01 | -.01 | .06 | -.03 | .88^***^ |

**Table S3c**

*Cross-domain correlations (Wave-1 associations)*

|  | Environmental-Health Concerns – Wave 1 | Socio-Political Concerns – Wave 1 | Concerns about Artificial Intelligence and Virtual Worlds – Wave 1 | Concerns about War and Economic Crisis – Wave 1 | Symptoms of depression – Wave 1 | Symptoms of anxiety– Wave 1 | Life satisfaction – Wave 1 |
| --- | --- | --- | --- | --- | --- | --- | --- |
| Attachment anxiety | .20*** | .22*** | .21*** | .16*** | .44*** | .37*** | -.18*** |
| Attachment avoidance | -.20*** | -.22*** | -.06* | -.21*** | .12*** | .18*** | -.28*** |
| Social support | .20*** | .17*** | .06 | .19*** | -.23*** | -.21*** | .33*** |
| Place identity | .07* | -.08** | .06* | .07* | -.10*** | -.06 | .23*** |
| Social engagement | .12*** | .16*** | .06 | .07* | .20*** | .20*** | .05 |
| Difficulties in emotion regulation | .17*** | .16*** | .15*** | .09** | .69*** | .69*** | -.26*** |
| Emotional attachment to place | .08** | -.03 | .06* | .09** | -.10** | -.05 | .22*** |
| Traditional place attachment | .00 | -0.08** | .14*** | .04 | -0.02 | -0.00 | .21*** |
| Active place attachment | .15*** | .05 | .07* | .10** | -.04 | .01 | .23*** |
| Diagnosis | .09** | .14*** | .03 | .06* | .35*** | .30*** | -0.17*** |
| Being in relationship | .10*** | .15*** | .03 | .11*** | -0.03 | .02 | .16*** |
| Socioeconomic status | .08** | .10** | .06 | .07* | -.06 | -.01 | .19*** |

**S4. Attrition analysis**

To examine systematic patterns of attrition, we compared incomplete responders (n = 732) to complete responders (n = 378) on key demographic variables and the main indicators used in the analysis. Complete responders were more likely to be women than men (χ²(1) = 18.73, p < .001) and were slightly older (M = 25.42 years, SD = 3.32) than non-completers (M = 24.32, SD = 3.74), t(1108) = 4.93, p < .001. Completers also reported slightly higher educational levels (M = 2.58, SD = .89) than non-completers (M = 2.55, SD = .85), U = 110,565.0, Z = −5.88, p < .001. Regarding baseline mental health, attrition was systematic: complete responders reported significantly fewer depressive symptoms (PHQ-9; M = 8.36, SD = 6.39) than non-completers (M = 9.60, SD = 6.30), U = 121,974.5, Z = −3.24, p = .001. Completers also scored lower on anxiety (GAD-7; M = 7.06, SD = 5.78 vs. M = 7.75, SD = 5.53), U = 127,220.0, Z = −2.20, p = .027, emotion-regulation difficulties (DERS-SF; M = 2.40, SD = .92 vs. M = 2.51, SD = .89), U = 127,964.5, Z = −2.05, p = .040, and attachment anxiety (ECR-Anxiety; M = 3.97, SD = 1.38 vs. M = 4.22, SD = 1.30), U = 124,090.5, Z = −2.82, p = .005. No attrition differences were observed for attachment avoidance (p = .364), any of the four crisis-concern scales (all p > .10), perceived social support, social engagement, traditional or active place attachment, or life satisfaction (all p > .05). To test the validity of the Missing at Random (MAR) assumption, we conducted a binary logistic regression model predicting attrition. The model explained R^2^Cox-and-Snell = .11 (11 %) of the variance in attrition over time. The results showed that attrition was significantly predicted by a combination of sociodemographic, relational, and individual factors: age (OR = 0.85, p < .001), gender (OR = 1.92, p < .001), education (OR = 0.90, p = .028), relationship status (being single; OR = 0.69, p = .018), and attachment anxiety (OR = 1.16, p = .017). Crucially, while univariate tests indicated differences in baseline distress, neither depressive symptoms (PHQ-9; p = .069) nor anxiety symptoms (GAD-7; p = .569) independently predicted attrition when these other factors were controlled for.

Hence, after including demographic variables in the model, any missing data in our key variables could be treated as missing at random (MAR; see Young and Johnson, 2015). Therefore, we used full information maximum likelihood to address missing data, as it provides unbiased parameter estimates under the MAR assumption (Enders and Bandalos, 2001). Taken together, these results suggest that attrition was systematic only with respect to demographic variables already accounted for in the analysis, and that the retained sample provides a valid basis for modeling dynamic mental-health trajectories in times of polycrisis.

**Table S4**

*Between-group factor analysis (MGCFA) model fit parameters with Satorra-Bentler correction*

|  | χ2 (df)^a^ | RMSEA | CFI | SRMR |
| --- | --- | --- | --- | --- |
| Environment–Health Concerns |  |  |  |  |
| Configural | 14.49 (15) | .01 | 1.00 | .02 |
| Metric | 32.98 (19) | .05 | .99 | .05 |
| Scalar | 47.99 (23) | .06 | .98 | .05 |
| Socio-Political Concerns |  |  |  |  |
| Configural | 41.64(15) | .07 | .98 | .03 |
| Metric | 55.17 (19) | .07 | .98 | .05 |
| Scalar | 63.73 (23) | .07 | .97 | .06 |
| AI/Virtual-Worlds Concerns |  |  |  |  |
| Configural | 17.87(15) | .03 | 1.00 | .03 |
| Metric | 22.29 (19) | .03 | 1.00 | .03 |
| Scalar | 32.67 (23) | .04 | .99 | .03 |
| War/Economic-Crisis Concerns |  |  |  |  |
| Configural | 257.80 (72) | .08 | .93 | .04 |
| Metric | 274.55 (80) | .08 | .92 | .05 |
| Scalar | 356.17 (88) | .09 | .90 | .06 |
| Anxiety Symptoms |  |  |  |  |
| Configural | 358.56(165) | .06 | .97 | .03 |
| Metric | 377.52(177) | .06 | .97 | .03 |
| Scalar | 409.35(189) | .06 | .96 | .03 |
| Depressive Symptoms |  |  |  |  |
| Configural | 618.14(294) | .05 | .96 | .04 |
| Metric | 644.49(310) | .05 | .96 | .05 |
| Scalar | 701.27(328) | .05 | .96 | .05 |
| Life Satisfaction |  |  |  |  |
| Configural | 105.10 (72) | .03 | 1.00 | .02 |
| Metric | 114.42 (80) | .03 | 1.00 | .03 |
| Scalar | 129.77 (88) | .03 | .99 | .03 |

*Note*: a - Satorra-Bentler correction

Table S5
 *Means and Standard Deviations for Wave-1 Indicators by Profile*

| Variable | Highly Worried, but Satisfied (n = 276) | Content & Carefree (n = 40) | Content & Mildly Concerned (n = 335) | Highly Distressed & Moderately Concerned (n = 298) | Severely Distressed & Highly Concerned (n = 161) | MANOVA  F/ *p* value | Significant comparisons  Tukey HSD test  p < .05 |
| --- | --- | --- | --- | --- | --- | --- | --- |
| Environment–Health Concerns | 3.97 (0.66) | 1.68 (0.66) | 2.87 (0.66) | 3.12 (0.66) | 4.23 (0.66) | 280.33 <.001 | 1 > 2, 1 > 3, 1 > 4, 1 < 5, 2 < 3, 2 < 4, 2 < 5, 3 < 4, 3 < 5, 4 < 5 |
| Socio-Political Concerns | 4.37 (0.74) | 1.92 (0.74) | 3.15 (0.74) | 3.55 (0.74) | 4.46 (0.74) | 231.78 <.001 | 1 > 2, 1 > 3, 1 > 4, 2 < 3, 2 < 4, 2 < 5, 3 < 4, 3 < 5, 4 < 5 |
| AI/Virtual-Worlds Concerns | 3.69 (0.79) | 1.82 (0.79) | 2.97 (0.79) | 3.03 (0.79) | 3.94 (0.79) | 109.80 <.001 | 1 > 2, 1 > 3, 1 > 4, 1 < 5, 2 < 3, 2 < 4, 2 < 5, 3 < 5, 4 < 5 |
| War/Economic-Crisis Concerns | 4.06 (0.57) | 1.83 (0.57) | 3.28 (0.57) | 3.35 (0.57) | 4.23 (0.57) | 258.91 <.001 | 1 > 2, 1 > 3, 1 > 4, 1 < 5, 2 < 3, 2 < 4, 2 < 5, 3 < 5, 4 < 5 |
| Anxiety Symptoms | 4.44 (5.63) | 2.62 (5.63) | 3.35 (5.63) | 11.36 (5.63) | 15.09 (5.63) | 582.92 <.001 | 1 > 3, 1 < 4, 1 < 5, 2 < 4, 2 < 5, 3 < 4, 3 < 5, 4 < 5 |
| Depressive Symptoms | 5.64 (6.35) | 3.87 (6.35) | 4.53 (6.35) | 13.75 (6.35) | 17.26 (6.35) | 541.51 <.001 | 1 > 2, 1 > 3, 1 < 4, 1 < 5, 2 < 4, 2 < 5, 3 < 4, 3 < 5, 4 < 5 |
| Life Satisfaction | 4.32 (1.18) | 4.08 (1.18) | 4.21 (1.18) | 3.58 (1.18) | 3.32 (1.18) | 30.196 <.001 | 1 > 4, 1 > 5, 2 > 5, 3 > 4, 3 > 5 |

**Table S6***Means and Standard Deviations for Wave-2 Indicators by Profile*

| Variable | Content & Carefree (n = 40) | Content & Mildly Concerned (n = 335) | Highly Worried, but Satisfied (n = 276) | Highly Distressed & Moderately Concerned (n = 298) | Severely Distressed & Highly Concerned (n = 161) |
| --- | --- | --- | --- | --- | --- |
| Environment–Health Concerns | 1.48 (0.64) | 2.92 (0.64) | 4.08 (0.64) | 2.87 (0.64) | 3.85 (0.64) |
| Socio-Political Concerns | 2.03 (0.69) | 3.20 (0.69) | 4.25 (0.69) | 3.08 (0.69) | 4.13 (0.69) |
| AI/Virtual-Worlds Concerns | 1.81 (0.70) | 2.95 (0.70) | 3.77 (0.70) | 2.95 (0.70) | 3.78 (0.70) |
| War/Economic-Crisis Concerns | 1.94 (0.59) | 3.32 (0.59) | 4.15 (0.59) | 3.32 (0.59) | 4.04 (0.59) |
| Anxiety Symptoms | 2.25 (5.79) | 2.90 (5.79) | 5.96 (5.79) | 9.99 (5.79) | 15.36 (5.79) |
| Depressive Symptoms | 3.66 (6.79) | 3.40 (6.79) | 6.51 (6.79) | 12.40 (6.79) | 18.33 (6.79) |
| Life Satisfaction | 3.89 (1.17) | 4.37 (1.17) | 4.36 (1.17) | 3.42 (1.17) | 3.80 (1.17) |

**Table S7***Means and Standard Deviations for Wave-3 Indicators by Profile*

| Variable | Content & Carefree (n = 40) | Content & Mildly Concerned (n = 335) | Highly Worried, but Satisfied (n = 276) | Highly Distressed & Moderately Concerned (n = 298) | Severely Distressed & Highly Concerned (n = 161) |
| --- | --- | --- | --- | --- | --- |
| Environment–Health Concerns | 1.64 (0.73) | 2.76 (0.73) | 4.01 (0.73) | 3.30 (0.73) | 3.82 (0.73) |
| Socio-Political Concerns | 1.54 (0.74) | 3.06 (0.74) | 4.13 (0.74) | 3.51 (0.74) | 4.04 (0.74) |
| AI/Virtual-Worlds Concerns | 1.44 (0.76) | 2.72 (0.76) | 4.03 (0.76) | 3.22 (0.76) | 4.04 (0.76) |
| War/Economic-Crisis Concerns | 1.95 (0.62) | 3.16 (0.62) | 4.18 (0.62) | 3.50 (0.62) | 4.15 (0.62) |
| Anxiety Symptoms | 3.82 (3.05) | 2.53 (3.05) | 4.25 (3.05) | 10.53 (3.05) | 17.82 (3.05) |
| Depressive Symptoms | 5.47 (3.83) | 3.03 (3.83) | 4.82 (3.83) | 12.31 (3.83) | 21.16 (3.83) |
| Life Satisfaction | 3.77 (1.25) | 4.36 (1.25) | 4.65 (1.25) | 3.76 (1.25) | 3.28 (1.25) |

**Table S8**

*Estimated transition probabilities among latent profiles*

| Wave 1 → Wave 2 | 1 | 2 | 3 | 4 | 5 |
| --- | --- | --- | --- | --- | --- |
| 1. Content & Carefree | .676 | .275 | .000 | .000 | .049 |
| 2. Content & Mildly Concerned | .078 | .803 | .001 | .117 | .001 |
| 3. Highly Worried, but Satisfied | .011 | .193 | .615 | .102 | .079 |
| 4. Highly Distressed & Moderately Concerned | .017 | .108 | .050 | .771 | .054 |
| 5. Severely Distressed & Highly Concerned | .013 | .000 | .088 | .072 | .827 |
| Wave 2 → Wave 3 | 1 | 2 | 3 | 4 | 5 |
| 1. Content & Carefree | .646 | .274 | .000 | .080 | .000 |
| 2. Content & Mildly Concerned | .042 | .843 | .041 | .074 | .000 |
| 3. Highly Worried, but Satisfied | .000 | .001 | .891 | .108 | .000 |
| 4. Highly Distressed & Moderately Concerned | .066 | .076 | .042 | .776 | .040 |
| 5. Severely Distressed & Highly Concerned | .000 | .000 | .062 | .177 | .760 |

**Supplementary Material S5. Sensitivity Analysis: Comparison of FIML and Multiple Imputation**

To address potential biases arising from longitudinal attrition, we conducted a sensitivity analysis comparing our primary estimation method—Full Information Maximum Likelihood (FIML)—with Multiple Imputation (MI). We generated 20 imputed datasets using chained equations and re-estimated the 5-class Latent Transition Analysis (LTA) model. We re-estimated solutions with two to six classes across all imputed datasets for each wave. As detailed in Table S10, the fit indices and class proportions for the MI-pooled models remained highly stable and nearly identical to the original FIML estimates. Across all waves, the five-profile solution maintained high classification quality, with Entropy values exceeding .80 in the MI-pooled results. This stability confirms that the separation between profiles—including the smaller "Content & Carefree" group—remains robust when accounting for missing data. We evaluated the raw indicator means for the 5-profile solution across all three waves under the MI-pooled estimation. The multivariate patterns defining each latent profile remained consistent with those in the primary FIML analysis.

Table S9
*Model Fit Indices and Class Proportions for 2–6 Latent Profile Solutions (Multiple Imputation Pooled Results)*

| Wave | Classes | Log-likelihood | AIC | BIC | aBIC | Entropy | Class proportions (%) |
| --- | --- | --- | --- | --- | --- | --- | --- |
| 1 | 2 | -14,136.62 | 28,317.25 | 28,427.52 | 28,357.64 | .825 | 59.8; 40.2 |
| 1 | 3 | -13,902.84 | 27,865.68 | 28,016.04 | 27,920.75 | .774 | 32.7; 29.9; 37.4 |
| 1 | 4 | -13,708.26 | 27,492.52 | 27,682.98 | 27,562.29 | .781 | 30.3; 15.8; 25.7; 28.2 |
| 1 | 5 | -13,592.13 | 27,276.25 | 27,506.81 | 27,360.70 | .805 | 3.5; 27.2; 29.8; 24.8; 14.8 |
| 1 | 6 | -13,463.94 | 27,035.88 | 27,306.29 | 27,134.77 | .794 | 3.3; 16.6; 30.0; 23.4; 16.4; 9.5 |
| 2 | 2 | -5,531.61 | 11,107.22 | 11,196.83 | 11,127.01 | .838 | 62.4; 37.6 |
| 2 | 3 | -5,418.55 | 10,897.09 | 11,019.29 | 10,924.08 | .838 | 34.1; 11.0; 54.9 |
| 2 | 4 | -5,336.01 | 10,748.01 | 10,902.79 | 10,782.19 | .835 | 7.2; 17.8; 47.2; 27.8 |
| 2 | 5 | -5,252.65 | 10,597.30 | 10,784.66 | 10,638.68 | .830 | 6.8; 22.4; 33.3; 18.2; 19.3 |
| 2 | 6 | -5,212.82 | 10,533.64 | 10,753.59 | 10,582.22 | .856 | 32.0; 0.7; 7.2; 24.2; 19.0; 16.9 |
| 3 | 2 | -4,953.28 | 9,950.56 | 10,037.13 | 9,967.33 | .817 | 62.8; 37.2 |
| 3 | 3 | -4,835.57 | 9,731.14 | 9,849.19 | 9,754.01 | .820 | 16.7; 29.9; 53.4 |
| 3 | 4 | -4,755.26 | 9,586.53 | 9,736.05 | 9,615.49 | .823 | 28.7; 12.1; 22.0; 37.2 |
| 3 | 5 | -4,699.39 | 9,490.78 | 9,671.78 | 9,525.83 | .843 | 6.5; 15.2; 36.3; 30.4; 11.7 |
| 3 | 6 | -4,658.10 | 9,424.21 | 9,636.69 | 9,465.36 | .866 | 8.1; 2.8; 29.9; 36.3; 13.2; 9.7 |

*Note.* AIC = Akaike information criterion; BIC = Bayesian information criterion; aBIC = sample-size adjusted BIC. Entropy ranges from 0 to 1, with higher values indicating clearer classification. Class proportions are reported as percentages.

Furthermore, we examined the diagonal transition probabilities to assess if attrition impacted the interpreted stability of the latent states. As presented in Table S10, the probabilities of remaining in the same profile (stability) remained highly consistent between FIML and MI methods.

Table S10.

*Comparison of Profile Stability (Diagonal Probabilities) between FIML and MI*

| Profiles | Wave 1 → Wave 2 (FIML) | Wave 1 → Wave 2 (MI) | Wave 2 → Wave 3 (FIML) | Wave 2 → Wave 3 (MI) |
| --- | --- | --- | --- | --- |
| Content & Carefree | .676 | .811 | .646 | .763 |
| Content & Mildly Concerned | .803 | .729 | .843 | .794 |
| Highly Worried but Satisfied | .615 | .657 | .891 | .793 |
| Highly Distressed | .771 | .766 | .776 | .810 |
| Severely Distressed | .827 | .774 | .760 | .777 |

*Note.* Values are diagonal transition probabilities (i.e., stability of remaining in the same latent profile across waves). FIML = full information maximum likelihood; MI = multiple imputation.

Table S11
*Multinomial logistic regression predicting Wave-1 latent-profile membership (Reference = Highly Distressed & Moderately Concerned): Odds Ratios and 95% Confidence Intervals*

| Predictor | Content & Carefree OR [95% CI] | Content & Mildly Concerned OR [95% CI] | Severely Distressed & Highly Concerned OR [95% CI] | Highly Worried but Satisfied OR [95% CI] |
| --- | --- | --- | --- | --- |
| Male gender (0 = female, 1 = male) | 6.82 [1.70, 27.29]** | 1.57 [0.93, 2.66] | 0.38 [0.20, 0.73]** | 0.40 [0.23, 0.70]** |
| Financial situation | 1.00 [0.63, 1.59] | 1.25 [0.98, 1.61] | 0.96 [0.73, 1.26] | 1.20 [0.97, 1.50] |
| Age (years) | 1.01 [0.88, 1.17] | 1.01 [0.93, 1.10] | 1.02 [0.92, 1.12] | 1.05 [0.97, 1.14] |
| Number of mental-health diagnoses | 0.81 [0.39, 1.69] | 0.56 [0.39, 0.81]** | 0.93 [0.70, 1.25] | 0.67 [0.49, 0.92]* |
| Emotional place attachment | 1.14 [0.66, 1.98] | 1.06 [0.76, 1.48] | 1.45 [0.98, 2.16] | 1.05 [0.74, 1.49] |
| Place identity | 1.02 [0.59, 1.77] | 1.02 [0.75, 1.39] | 0.69 [0.50, 0.97]* | 1.11 [0.81, 1.52] |
| Emotion-regulation difficulties | 0.07 [0.03, 0.17]*** | 0.18 [0.12, 0.27]*** | 3.99 [1.94, 8.19]*** | 0.21 [0.14, 0.32]*** |
| Attachment anxiety | 0.54 [0.34, 0.86]** | 0.58 [0.42, 0.79]*** | 2.69 [1.07, 6.80]* | 0.85 [0.63, 1.16] |
| Attachment avoidance | 0.67 [0.36, 1.24] | 1.01 [0.67, 1.52] | 1.15 [0.53, 2.51] | 0.67 [0.46, 0.97]* |
| Social support | 0.98 [0.87, 1.11] | 1.02 [0.95, 1.09] | 1.09 [0.98, 1.22] | 1.14 [1.06, 1.22]*** |
| Social engagement | 0.12 [0.01, 1.06] | 0.47 [0.26, 0.84]* | 0.51 [0.25, 1.03] | 1.13 [0.66, 1.91] |
| Traditional place attachment | 0.66 [0.41, 1.07] | 1.00 [0.81, 1.25] | 0.96 [0.73, 1.25] | 0.90 [0.73, 1.11] |
| Active place attachment | 0.67 [0.46, 0.98]* | 0.80 [0.63, 1.02] | 0.95 [0.71, 1.28] | 1.01 [0.81, 1.27] |
| Size of place of residence | 1.13 [0.80, 1.60] | 0.99 [0.83, 1.18] | 1.03 [0.80, 1.33] | 0.92 [0.76, 1.11] |
| Level of education | 0.82 [0.45, 1.49] | 0.93 [0.66, 1.30] | 1.22 [0.83, 1.77] | 0.97 [0.70, 1.35] |
| In a romantic relationship | 2.21 [0.62, 7.81] | 0.87 [0.49, 1.55] | 1.55 [0.80, 3.00] | 0.97 [0.56, 1.68] |

Note. OR = odds ratio; CI = confidence interval.
 *p* < .05; **p* < .01; ***p* < .001.

**Table S12**
*Predictors of Wave-1 Latent-Profile Membership: Odds Ratios (95% CIs) (Reference = Severely Distressed & Highly Concerned)*

| Predictor | Content & Carefree OR [95% CI] | Content & Mildly Concerned OR [95% CI] | Highly Distressed & Moderately Concerned OR [95% CI] | Highly Worried but Satisfied OR [95% CI] |
| --- | --- | --- | --- | --- |
| Male gender (0 = female, 1 = male) | 18.05 [4.12, 79.11]*** | 4.16 [2.03, 8.54]*** | 2.65 [1.36, 5.13]** | 1.05 [0.50, 2.23] |
| Financial situation | 1.04 [0.62, 1.75] | 1.31 [0.94, 1.82] | 1.04 [0.79, 1.37] | 1.25 [0.91, 1.73] |
| Age (years) | 1.00 [0.85, 1.17] | 1.00 [0.89, 1.11] | 0.98 [0.89, 1.08] | 1.03 [0.93, 1.15] |
| Number of mental-health diagnoses | 0.87 [0.40, 1.90] | 0.60 [0.39, 0.93]* | 1.07 [0.80, 1.43] | 0.72 [0.49, 1.07] |
| Emotional place attachment | 0.79 [0.42, 1.47] | 0.73 [0.47, 1.14] | 0.69 [0.46, 1.02] | 0.72 [0.46, 1.13] |
| Place identity | 1.47 [0.81, 2.66] | 1.47 [1.01, 2.13]* | 1.44 [1.04, 2.00]* | 1.60 [1.11, 2.29]* |
| Emotion-regulation difficulties | 0.02 [0.00, 0.05]*** | 0.05 [0.02, 0.10]*** | 0.25 [0.12, 0.51]*** | 0.05 [0.02, 0.12]*** |
| Attachment anxiety | 0.20 [0.07, 0.56]** | 0.21 [0.08, 0.56]** | 0.37 [0.15, 0.94]* | 0.32 [0.12, 0.84]* |
| Attachment avoidance | 0.58 [0.22, 1.51] | 0.87 [0.38, 1.99] | 0.87 [0.40, 1.90] | 0.58 [0.26, 1.31] |
| Social support | 0.90 [0.77, 1.04] | 0.93 [0.83, 1.04] | 0.91 [082, 1.02] | 1.04 [0.93, 1.16] |
| Social engagement | 0.23 [0.02, 2.22] | 0.92 [0.42, 2.06] | 1.96 [0.97, 3.96] | 2.21 [1.06, 4.61]* |
| Traditional place attachment | 0.69 [0.41, 1.17] | 1.05 [0.78, 1.41] | 1.04 [0.80, 1.36] | 0.94 [0.70, 1.26] |
| Active place attachment | 0.71 [0.46, 1.09] | 0.84 [0.61, 1.15] | 1.05 [0.78, 1.41] | 1.06 [0.78, 1.34] |
| Size of place of residence | 1.10 [0.73, 1.65] | 0.96 [0.74, 1.26] | 0.97 [0.75, 1.33] | 0.89 [0.68, 1.18] |
| Level of education | 0.67 [0.35, 1.30] | 0.76 [0.49, 1.18] | 0.82 [0.57, 1.20] | 0.80 [0.52, 1.23] |
| In a romantic relationship | 1.42 [0.37, 5.51] | 0.56 [0.27, 1.19] | 0.65 [0.33, 1.25] | 0.63 [0.30, 1.30] |

*Note.* OR = odds ratio; CI = confidence interval.
 *p* < .05; **p* < .01; ***p* < .001.

Table S13
*Predictors of Wave-1 Latent-Profile Membership: Odds Ratios (95% CIs)* *(Reference = Highly Worried but Satisfied*)

| Predictor | Content & Carefree OR [95% CI] | Content & Mildly Concerned OR [95% CI] | Severely Distressed & Highly Concerned OR [95% CI] | Highly Distressed & Moderately Concerned OR [95% CI] |
| --- | --- | --- | --- | --- |
| Male gender (0 = female, 1 = male) | 17.17*** [4.39, 67.17] | 3.96*** [2.38, 6.58] | 0.95 [0.45, 2.00] | 2.52** [1.44, 4.41] |
| Financial situation | 0.83 [0.54, 1.29] | 1.04 [0.84, 1.30] | 0.80 [0.58, 1.10] | 0.83 [0.67, 1.03] |
| Age (years) | 0.96 [0.85, 1.10] | 0.96 [0.90, 1.01] | 0.97 [0.88, 1.07] | 0.95 [0.88, 1.03] |
| Number of mental-health diagnoses | 1.20 [0.57, 2.55] | 0.83 [0.54, 1.29] | 1.39  [0.93, 2.08] | 1.49* [1.08, 2.04] |
| Emotional place attachment | 1.09 [0.63, 1.89] | 1.01 [0.71, 1.45] | 1.38 [0.88, 2.17] | 0.95 [0.67, 1.36] |
| Place identity | 0.92 [0.53, 1.61] | 0.92 [0.67, 1.26] | 0.63* [0.44, 0.89] | 0.90 [0.66, 1.24] |
| Emotion-regulation difficulties | 0.31* [0.12, 0.82] | 0.85 [0.55, 1.31] | 18.84*** [8.32, 42.66] | 4.72*** [3.12, 7.15] |
| Attachment anxiety | 0.64* [0.43, 0.93] | 0.68*** [0.55, 0.83] | 3.15* [1.18, 8.36] | 1.17 [0.86, 1.59] |
| Attachment avoidance | 1.00 [0.58, 1.73] | 1.51** [1.14, 2.01] | 1.72 [0.74, 3.99] | 1.50* [1.03, 2.18] |
| Social support | 0.86* [0.77, 0.97] | 0.89*** [0.83, 0.95] | 0.96 [0.86, 1.07] | 0.88*** [0.82, 0.94] |
| Social engagement | 0.10* [0.01, 0.93] | 0.42** [0.26, 0.68] | 0.45* [0.22, 0.95] | 0.89 [0.52, 1.51] |
| Traditional place attachment | 0.74 [0.47, 1.16] | 1.12 [0.94, 1.33] | 1.07 [0.80, 1.43] | 1.11 [0.90, 1.38] |
| Active place attachment | 0.67* [0.47, 0.95] | 0.79** [0.66, 0.95] | 0.94 [0.70, 1.27] | 0.99 [0.79, 1.24] |
| Size of place of residence | 1.23 [0.88, 1.72] | 1.08 [0.93, 1.25] | 1.12 [0.88, 1.42] | 1.09 [0.90, 1.31] |
| Level of education | 0.84 [0.47, 1.49] | 0.95 [0.73, 1.24] | 1.25 [0.79, 1.98] | 1.03 [0.74, 1.43] |
| In a romantic relationship | 2.27 [0.64, 8.00] | 0.90 [0.52, 1.55] | 1.59 [0.81, 3.12] | 1.03 [0.60, 1.78] |

*Note.* OR = odds ratio; CI = confidence interval; * p < .05; ** p < .01; *** p < .001.

Table S14
*Predictors of Wave-1 Latent-Profile Membership: Odds Ratios (95% CIs) and Significance (Reference = Content & Carefree)*

| Predictor | Content & Mildly Concerned OR [95% CI] | Severely Distressed & Highly Concerned OR [95% CI] | Highly Distressed & Moderately Concerned OR [95% CI] | Highly Worried but Satisfied OR [95% CI] |
| --- | --- | --- | --- | --- |
| Male gender (0 = female, 1 = male) | 0.23 [0.06, 0.90]* | 0.06 [0.01, 0.24]*** | 0.15 [0.04, 0.59]** | 0.06 [0.02, 0.23]*** |
| Financial situation | 1.25 [0.82, 1.92] | 0.96 [0.57, 1.60] | 1.00 [0.63, 1.58] | 1.20 [0.78, 1.86] |
| Age (years) | 1.00 [0.88, 1.13] | 1.00 [0.86, 1.17] | 0.99 [0.86, 1.13] | 1.04 [0.91, 1.18] |
| Number of mental-health diagnoses | 0.69 [0.32, 1.49] | 1.15 [0.53, 2.52] | 1.23 [0.59, 2.58] | 0.83 [0.39, 1.76] |
| Emotional place attachment | 0.93 [0.55, 1.58] | 1.27 [0.68, 2.39] | 0.88 [0.51, 1.52] | 0.92 [0.53, 1.60] |
| Place identity | 1.00 [0.58, 1.73] | 0.68 [0.38, 1.23] | 0.98 [0.57, 1.70] | 1.09 [0.62, 1.90] |
| Emotion-regulation difficulties | 2.74 [1.06, 7.04]* | 60.93 [18.40, 200.75]*** | 15.26 [5.78, 40.25]*** | 3.23 [1.22, 8.53]* |
| Attachment anxiety | 1.06 [0.74, 1.53] | 4.96 [1.79, 13.77]** | 1.84 [1.17, 2.91]** | 1.57 [1.08, 2.30]* |
| Attachment avoidance | 1.51 [0.87, 2.64] | 1.73 [0.66, 4.50] | 1.50 [0.81, 2.79] | 1.00 [0.58, 1.74] |
| Social support | 1.03 [0.92, 1.15] | 1.11 [0.96, 1.29] | 1.02 [0.90, 1.15] | 1.16 [1.03, 1.31]* |
| Social engagement | 4.06 [0.44, 37.93] | 4.40 [0.45, 42.90] | 8.61 [0.95, 78.29] | 9.70 [1.08, 87.45]* |
| Traditional place attachment | 1.51 [0.95, 2.40] | 1.44 [0.85, 2.44] | 1.51 [0.93, 2.44] | 1.35 [0.86, 2.13] |
| Active place attachment | 1.19 [0.83, 1.68] | 1.42 [0.92, 2.19] | 1.49 [1.02, 2.17]* | 1.50 [1.06, 2.14]* |
| Size of place of residence | 0.87 [0.63, 1.21] | 0.91 [0.61, 1.36] | 0.88 [0.62, 1.25] | 0.81 [0.58, 1.13] |
| Level of education | 1.14 [0.64, 2.01] | 1.49 [0.77, 2.90] | 1.23 [0.67, 2.23] | 1.19 [0.67, 2.12] |
| In a romantic relationship | 0.40 [0.12, 1.34] | 0.70 [0.18, 2.71] | 0.45 [0.13, 1.61] | 0.44 [0.13, 1.50] |

*Note.* OR = odds ratio; CI = confidence interval; * p < .05; ** p < .01; *** p < .001.

**Table S15**
*Odds ratios (OR) [95 % CI] for moving from each Wave 1 profile to a different Wave 2 profile.*

| Predictor | From Profile | To Profile | OR [95 % CI] |
| --- | --- | --- | --- |
| Male gender (0 = female, 1 = male) | Highly Distressed & Moderately Concerned | Highly Worried but Satisfied | 4.20 [1.08, 16.34] |
|  | Highly Worried but Satisfied | Highly Distressed & Moderately Concerned | 0.24 [0.06, 0.93] |
| Mental-health diagnoses | Highly Distressed & Moderately Concerned | Content & Mildly Concerned | 0.50 [0.26, 0.95] |
|  | Content & Mildly Concerned | Highly Distressed & Moderately Concerned | 2.01 [1.06, 3.82] |
|  | Content & Mildly Concerned | Highly Worried but Satisfied | 2.61 [1.12, 6.12] |
|  | Highly Worried but Satisfied | Content & Mildly Concerned | 0.38 [0.16, 0.90] |
| Attachment avoidance | Highly Distressed & Moderately Concerned | Content & Mildly Concerned | 0.51 [0.32, 0.79] |
|  | Content & Mildly Concerned | Highly Distressed & Moderately Concerned | 1.98 [1.27, 3.09] |
| Social support | Highly Distressed & Moderately Concerned | Content & Carefree | 1.26 [1.01, 1.57] |
|  | Severely Distressed & Highly Concerned | Content & Carefree | 1.38 [1.06, 1.81] |
|  | Content & Mildly Concerned | Content & Carefree | 1.30 [1.03, 1.63] |
|  | Highly Worried but Satisfied | Content & Carefree | 1.33 [1.03, 1.73] |
|  | Content & Carefree | Highly Distressed & Moderately Concerned | 0.80 [0.64, 0.99] |
|  | Content & Carefree | Severely Distressed & Highly Concerned | 0.72 [0.55, 0.95] |
|  | Content & Carefree | Content & Mildly Concerned | 0.77 [0.61, 0.97] |
|  | Content & Carefree | Highly Worried but Satisfied | 0.75 [0.58, 0.98] |

*Note.* Only statistically significant transition effects (p < .05) are presented.

Table S16. Significant Predictors of Profile Transitions (Wave 2 → Wave 3)
*Odds ratios (OR) [95 % CI] for moving from each Wave 2 profile to a different Wave 3 profile.*

| Predictor | From Profile | To Profile | OR [95 % CI] |
| --- | --- | --- | --- |
| Mental-health diagnoses |  |  |  |
|  | Highly Distressed & Moderately Concerned | Content & Carefree | 5.81 [1.11, 30.34]] |
|  | Severely Distressed & Highly Concerned | Content & Carefree | 11.36 [1.43, 90.40] |
|  | Highly Worried but Satisfied | Content & Carefree | 9.28 [1.23, 69.80] |
|  | Content & Carefree | Highly Distressed & Moderately Concerned | 0.17 [0.03, 0.90] |
|  | Content & Carefree | Severely Distressed & Highly Concerned | 0.09 [0.01, 0.70] |
|  | Content & Carefree | Highly Worried but Satisfied | 0.11 [0.01, 0.81] |
| Place identity | Content & Carefree | Highly Distressed & Moderately Concerned | 0.27 [0.08, 0.85] |
|  | Highly Distressed & Moderately Concerned | Content & Carefree | 3.73[1.17, 11.88] |
|  | Highly Distressed & Moderately Concerned | Content & Mildly Concerned | 2.63 [1.04, 6.67] |
|  | Content & Mildly Concerned | Highly Distressed & Moderately Concerned | 0.38 [0.15, 0.96] |

*Note.* Only statistically significant transition effects (p < .05) are presented.
